# Enhancing Detection of Message Intents in a Mobile Health Smoking-Cessation Intervention Using Large Language Model Fine-Tuning, Data Downsampling, and Error Correction: Algorithm Development and Validation

**DOI:** 10.2196/83437

**Published:** 2026-03-09

**Authors:** Shagoto Rahman, Cornelia (Connie) Pechmann, Ian G Harris

**Affiliations:** 1 Department of Computer Science, University of California, Irvine, Irvine, CA, 92697, United States, 1 9492334120; 2 The Paul Merage School of Business, University of California, Irvine, Irvine, CA, United States

**Keywords:** smoking cessation, intent detection, data imbalance, large language model, human annotation error analysis, chatbot

## Abstract

**Background:**

Although smoking-cessation aids such as support groups and nicotine replacement therapy (NRT) can help people quit, quit rates remain low. Mobile health interventions can boost accessibility and engagement, especially with NRT, but require ongoing effort to deliver timely responses. Accurate intent detection is crucial for identifying user needs and delivering timely, appropriate chatbot responses. Recent large language model advancements in natural language processing and artificial intelligence (AI) have shown promise. However, these systems often struggle with many intent categories, complex language, and imbalanced data, reducing recognition accuracy.

**Objective:**

The main goal of this study was to develop an AI tool, a large language model that could accurately detect people’s message intents, despite dataset imbalances and complexities. In our application, the messages came from a smoking-cessation support-group intervention and often involved the use of NRT provided as part of that intervention.

**Methods:**

We consistently used a state-of-the-art public domain large language model, Llama-3 8B (8 billion parameters) from Meta. First, we used the model off-the-shelf. Second, we fine-tuned it on our annotated dataset with 25 intent categories. Third, we also downsampled the predominant intent category to reduce model bias. Finally, we combined downsampling with corrected human annotations, creating a cleaned dataset for a new round of fine-tuning.

**Results:**

Without fine-tuning, the model achieved unweighted and weighted *F*_1_-scores (overall performance) of 0.41 and 0.38, respectively, on the downsampled corrected test dataset, and 0.29 and 0.35 on the full test dataset. Fine-tuning improved performance to 0.77 and 0.80 on the downsampled corrected dataset, and 0.72 and 0.86 on the full dataset. Fine-tuning with downsampling attained the best *F*_1_-scores, 0.88 and 0.91 on the downsampled corrected dataset, though performance dropped on the full test dataset (0.58 unweighted, 0.66 weighted) due to the predominance of the off-topic intent category, while unweighted recall remained high (0.80). The final method combining fine-tuning, downsampling, and error correction achieved 0.86 unweighted and 0.90 weighted *F*_1_-scores on the downsampled corrected dataset, and 0.57 and 0.65 on the full dataset with unweighted recall improving to 0.82.

**Conclusions:**

Large language models performed poorly without fine-tuning, highlighting the need for domain-specific training. Even with fine-tuning, performance was limited by a highly imbalanced dataset. Downsampling before fine-tuning moderately improved performance but still left room for improvement and concerns about dataset noise. A careful review of model-human disagreement cases helped identify human annotation errors. After error correction, the method without error correction still achieved slightly higher precision and *F*_1_-score on the corrected test dataset. While error correction slightly improved recall on noisy data, automated downsampling alone may be sufficient, making manual correction a more resource-intensive option with limited added benefit.

## Introduction

### Background

Although most smokers wish to stop smoking, cessation rates remain low in part because of the insufficient use of the cessation aids [1-3]. The use of mobile health interventions to aid people in quitting smoking has become commonplace [4,5], including SMS text messaging programs [6,7] and quit-smoking apps [4,8]. As traditional in-person support groups significantly improve quit rates [9,10], online groups have emerged to help overcome usage barriers related to accessibility and convenience [11]. Mobile health smoking-cessation support groups offer considerable promise; however, they require ongoing, resource-intensive efforts to provide timely, 24/7 responses that sustain participant engagement. Research has consistently demonstrated that active involvement in online support groups leads to improved outcomes [12,13], and studies also show that low engagement is often linked to dropouts [14].

Chatbots can enhance support-group member engagement and minimize dropout rates by providing continuous, cost-effective support [15-17]. Accurate intent detection, defined as the precise and consistent classification of a user’s communication goal from their input message, is a leading determinant of chatbot effectiveness. Accurate intent detection can improve user engagement by providing the inputs needed to the system to provide personalized, accurate, and timely support. It can also minimize the need for human intervention. With accurate intent detection, a chatbot can engage users by actively responding to messages with relevant content, addressing their concerns, and delivering evidence-based health information and support. For example, if a user indicates they have failed to quit smoking, the chatbot can identify this particular intent and suggest ways to overcome the challenge.

Previous chatbot studies have primarily focused on 1-to-1 conversations between chatbot and user, for example, for mental health or substance abuse screening [18] or interventions [19]. We study conversations in support groups, often involving several group members, which have unique properties compared to 1-to-1 conversations including multiparty dialogue, interruptions, subgroup discussions, and incomplete messaging leading to exceptionally unstructured and noisy data [20]. In addition, research has been done to detect message intents in medical settings, for example, online forums or office visits, but most involved limited intent categories which limited their ability to fully capture the message content [21,22].

Moreover, data imbalance is particularly common in the health domain because sufficient data is rarely available for all intent categories, yet ones with fewer samples can be critically important [23,24]. For example, an intent category such as overdose may have very few messages, but it represents a critical concern. Most prior studies did not properly ensure that the samples or exemplars were sufficiently balanced across intent categories, possibly leading to suboptimal model performance. Data downsampling, which involves reducing the number of samples from highly dominant majority categories to achieve a more balanced category distribution, can play a vital role. This approach can help the classifying model learn to detect all intent categories comparably, including those that are rare but critically important.

Large language models can deliver state-of-the-art performance in recognizing message intents because they are trained on vast amounts of data. As a result, these models can grasp the deeper meaning of text and often replicate human-like understanding. However, for highly specific classification tasks, large language models must be fine-tuned on domain-specific datasets where humans have annotated the message intents for this fine-tuning phase. Since data annotation is primarily done by humans, it often contains errors due to message ambiguity, human misunderstanding, carelessness, bias, or other factors. Manually reviewing the entire dataset to find human annotation errors is costly and time-consuming. Instead, focusing only on the data points where the model and human annotators disagree on the user’s message intent can save time and effort while effectively identifying annotation issues.

In this study, we demonstrate common challenges and solutions in using large language models for mobile health interventions. We implement a state-of-the-art, public domain, large language model called Llama-3 8B (8 billion parameters) from Meta to detect the message intents in a mobile health smoking-cessation intervention. Our study method consists of 4 parts. Part 1 involves intent detection using the Llama-3 8B large language model off-the-shelf, without fine-tuning. Part 2 fine-tunes the model on a domain-specific dataset which human annotators annotated with what they believed to be the message intents. Part 3 combines fine-tuning with downsampling of the majority (predominant) intent category, which was found to bias the model and weaken its performance at intent recognition. Part 4 adds correction of human error in intent annotation to the fine-tuning and downsampling. The rest of this paper is organized as follows: We elaborate on prior work, discuss our methods, report our experimental results, summarize the principal findings and limitations, and state future research directions and a conclusion.

### Prior Work

#### Conversational Agents in Health Care

Conversational health care agents have become important resources for providing quick and easily accessible medical assistance to patients. Recent research suggests that by harnessing advancements in natural language processing, they can provide personalized patient support and alleviate the excessive workload of many health care professionals. Babu and Boddu [25] created a BERT (Bidirectional Encoder Representations From Transformers)-based medical conversational agent to provide responses to people seeking medical advice, appointments, or information about specific medical conditions. The BERT model was fine-tuned on public databases such as MIMIC-III (Medical Information Mart for Intensive Care-III), PubMed, and a COVID-19 dataset totaling 11,000 samples of medical questions. The conversational agent integrated these extracted datasets with the ongoing conversation context and produced relevant and personalized feedback. When compared to baseline models such as LSTM (long short-term memory), SVM (support vector machine), and Bi-LSTM (bidirectional long short-term memory), the chatbot demonstrated superior performance, attaining 98% accuracy along with high precision, recall, and *F*_1_-scores combining precision and accuracy. However, the system demanded high computational power and suffered from data bias and interpretability issues.

Yu and McGuinness [26] developed a conversational agent by combining a fine-tuned DialoGPT model and ChatGPT to assist mental health conversations. The authors sought to address current model limitations in delivering proper and safe therapeutic responses due to their lack of training on specialized data. So, authors fine-tuned the DialoGPT model on a domain-specific dataset focused on cognitive behavioral therapy and integrated it with ChatGPT’s dynamic conversational capabilities. This hybrid conversational agent system was compared to conventional rule-based models and large language models on metrics such as BLEU (Bilingual Evaluation Understudy), which assesses the match between the model output and human-written text. Inputs from mental health professionals and patients were also considered. The results demonstrated that the hybrid system outperformed others in terms of conversational quality, relevance, and emotional resonance.

Zamani et al [27] sought to address the limitations of rule-based chatbots as mental health conversational agents, including lack of response creativity and low user acceptability. They also sought to address the risks of large language models in terms of generating harmful texts and the limitations of data imbalance. The authors evaluated the impact of novel text augmentation techniques to improve the message intent detection. Their study demonstrated that increasing the training dataset through augmentation could boost model accuracy by contributing to dataset diversity. The study incorporated easy data augmentation (EDA), synonym replacement using WordNet, embedding-based replacement, and context-aware substitutions. Model performance improved markedly through data augmentation, with up to a 26.4% increment in the average intent detection score.

#### Large Language Models for Intent Detection in Health Care

Grasping the speaker’s message intent is essential for facilitating smooth and meaningful human interaction. With recent advancements in natural language processing and deep learning, various recent studies have demonstrated success in detecting the subtle intents behind spoken expressions. Aftab et al [28] developed an intent classification method for health care conversational agents by incorporating Bayesian LSTMs. They used an open-source dataset containing 6661 text utterances corresponding to 25 unique medical symptom-related intents with a relatively balanced distribution of intent categories. Their method attained an impressive 99.4% accuracy in recognizing message intents on the test dataset. Moreover, the authors used Shannon entropy from several random model runs to measure uncertainty, which helped the system distinguish between familiar and unfamiliar inputs. Additionally, to estimate model uncertainty, they used Monte Carlo dropout during inference. As a result, the chatbot handled uncertain predictions more appropriately, allowing it to generate safer, less harmful responses.

Zhang et al [29] developed Conco-ERNIE, an innovative model designed to enhance user intent detection in complex medical queries by permitting single intent, multi-intent, and implicit intent recognition. The model used a special feature extractor called ERNIE with an added layer to help it understand and annotate important parts of medical questions. The authors incorporated the Apriori algorithm to find patterns in how medical concepts related to each other, allowing it to better detect intents when the uttered meanings were unclear. Moreover, they introduced an attention-based method that combined the meaning of words in a query with the patterns of related medical concepts. This helped the model focus better on important information and ignore irrelevant details. The authors tested their method on a dataset of real medical questions, and it showed clear improvements in detecting multiple and hidden user intents. Overall, the model achieved a score of 89.4% in test data, much higher than other popular methods. The authors then added their model Conco-ERNIE to a real health care chatbot linked to a large medical knowledge base.

Sakurai and Miyao [30] studied large language models for intent detection in conversations involving donation requests. The study examined conversations where the responder expressed criticism of donations or sought to make donors feel guilty. The authors primarily studied GPT-4 in this context and found it often misidentified critical, sarcastic, or negative affective responses as motivational or neutral. GPT-4 was tested on both real and artificially created conversations, and its predictions about intent were compared to human-annotated intents. Humans were able to detect critical and other negative messages, but GPT-4 often misunderstood them as encouraging or motivational. Overall, this study found that a state-of-the-art large language model struggled to detect subtle intents in negative speech.

### Our Contributions

In this paper, we demonstrate that a current state-of-the-art large language model cannot adequately detect subtle intents expressed by participants in a smoking-cessation intervention; for example, the model struggles to decipher substantively different messages about the study-provided nicotine replacement therapy (NRT). The methodological novelty of our work lies in systematically addressing highly imbalanced, domain-specific data for smoking-cessation text classification using the Llama-3 8B model. We illustrate the specific steps that can be taken, using existing analytical methods, to improve the model’s intent detection. We show vast improvements by fine-tuning the off-the-shelf model using a domain-specific dataset with human annotations of 25 message intents. We show additional substantial improvements by downsampling (reducing excess examples of) an overly dominant intent category and by correcting human annotation errors identified by examining model-human disagreement in intent classification. In sum, we show that large language models can perform very poorly in domain-specific contexts like ours but can be massively improved with current analytical approaches. This approach improves efficiency and robustness, providing insights for future work such as multi-intent detection, probability calibration, and translation of technical performance into operational impact.

## Methods

### Overview

**Figure 1.**
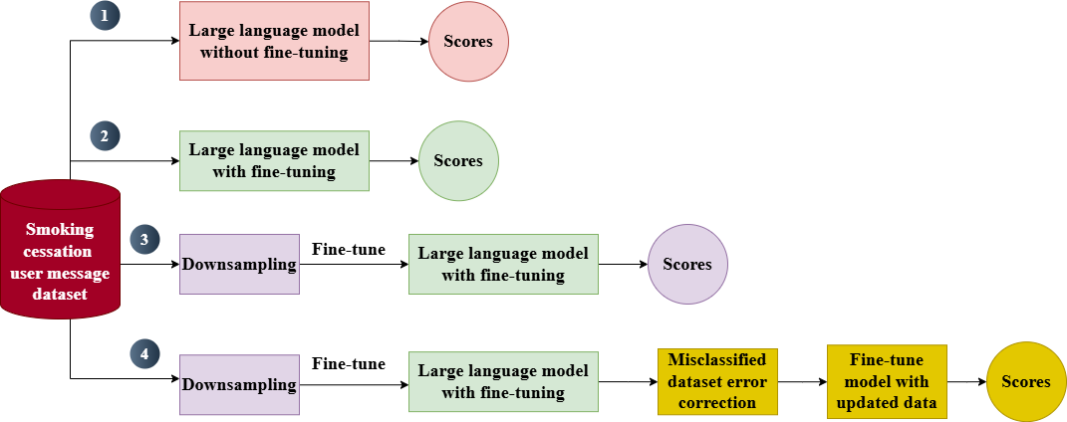
Overview of the 4 large language model training methods used for intent detection in this study (United States).

Figure 1 presents our method for training a large language model for the detection of message intents. It involved four stages: (1) large language model without fine-tuning, where the model predicted intents without any domain-specific training dataset solely using its own knowledge; (2) large language model with fine-tuning, where the model was fine-tuned on an intent-annotated dataset to improve performance; (3) large language model with fine-tuning and downsampling, which addressed category imbalance by reducing the sample size of a majority (dominant) intent category, which was off-topic or nondomain related, before fine-tuning; and (4) large language model with fine-tuning, downsampling, and human annotation error correction, where model-human disagreements were reviewed for potential human annotation errors, corrected if necessary, and used to retrain the model. This stepwise approach allowed for progressive enhancement in classification accuracy, with each stage addressing specific limitations of the previous one.

### Dataset

For this study, we use all the user messages from the Tweet2Quit mobile health smoking-cessation intervention [17]. All duplicated messages have been properly identified and removed, ensuring the dataset is clean and free from leakage. Tweet2Quit is a social media–based intervention to assist people with quitting smoking. Participants were assigned to private online support groups of 20 members who engaged in peer support discussions about smoking cessation. The dataset contained more than 82,000 messages gathered from 45 support groups participating in 2 clinical trials conducted between 2012‐2014 and 2016‐2019. Each support group remained active for 3 months and generated approximately 1822 messages on average. The messages were created by individuals actively working to quit smoking and then stay quit and, as a result, contained domain-rich, real-world interactions regarding quitting and the free NRT, or NRT (nicotine patches, gum, and lozenges) provided as a quitting aid.

A team of 25 trained research assistants, mostly US undergraduates from diverse majors, with a few international participants, annotated the intent behind each message to produce a comprehensive annotated corpus. The assistants were trained on intent definitions, keywords, and examples, then practiced on a set of messages until they attained the required ≥80% accuracy. Initial in-person training meetings transitioned to Microsoft Teams due to team expansion and COVID-19, with weekly sessions during training and biweekly discussions thereafter to resolve difficult cases. Each message was independently annotated by two assistants, with discrepancies resolved by a third expert adjudicator.

A total of 25 intent categories were identified while annotating the messages. The topics either were clinically related to smoking cessation (eg, nrt_howtouse, nrt_overdose, and nrt_mouthirritation), reflected social support for quitting (eg, greetings, support), or were off-topic (eg, small talk, weather, news). Table 1 presents the 25 message intent categories identified in the dataset, along with their definitions and representative examples. The annotation demonstrated high reliability, with a Cohen κ score of 0.93 overall, and agreement between specific annotators ranging from 87.3% to 97.2% across intent categories. The off-topic intent category was used to indicate irrelevant messages unrelated to smoking cessation or support group bonding. It alone accounted for 56% of the dataset, which indicated significant category imbalance.

**Table 1.** Definitions and examples of smoking-cessation message intents used in this intent detection study (United States, 2012‐2014 and 2016‐2019).

Intent category	Definition	Examples
cigsmell	Complains about cigarette smell	Cigarette smoke and cigarette smokers stink to me now. I can smell people smoking a mile away now. It's gross to me now.
cravings	Discusses craving/wanting/temptation/urge/trigger to smoke or how to fight/avoid craving	It's hard because all I want to do is reach over and get a cigarette and smoke. My urge is in the evening watching TV I usually occupy my mind with crochet that's all I really have.
ecigs	States something about ecigs, vape, or liquid or juice	Is anyone going to try vaping to help quit? The non-nicotine juice of course. I've been debating. I do like the Vape pen.
fail	Admits to smoking a cigarette	I have to admit I slipped today and smoked a cigarette. Mornings I'm a chain smoker.
greetings	Says a greeting, eg, hi	Good afternoon! How's everyone doing today? Good morning!
health	States something about the relation between smoking and health	My breathing is much better and I don't never want to smoke a cigarette again. To be able to breathe better.
nrt_dontwork	States NRT^a^ doesn’t work, don’t like, no longer needed	Since the gum and patches don't seem to work on my boring days I have struggled. This gum is a no go for me. I called the smoke shop in my town.
nrt_dreams	States they either sleep with the patch or try not to do so; often discusses dreams	I'm sleeping with patch on. Have the craziest most vivid dreams. Yes! Very lucid dreams. I had to stop sleeping with them on.
nrt_howtouse	Asks question or gives info about how to use NRT in general, eg, dose	How often should I use the lozenges? So your not suppose to constantly chew the gum right? Suppose to just let it lay in your gums once broken up right?
nrt_itworks	States reason why NRT used, eg, it works, stops craving	I'm using 1 patch a day and chewing the gum as needed! Both help immensely with cravings! I have stepped down to the 7mg patch!!!! Yea!!!!!
nrt_mouthirritation	States NRT gum/lozenge causes bad taste, throat gum irritation or burning, or spicy sensation	I toss the gum when it starts burning my mouth or throat. Don't like the gum I'm having a hard time chewing it. Burns my mouth a little.
nrt_nauseous	States NRT makes them nauseous or gag or sickly	Don't like the gum makes me nauseous. Using the #14 patch. Not using gum it makes me sick.
nrt_od	States NRT is too strong, feels like overdose	I am going to move to the step 2 patch. The 21mg patches are making me sweat and feel bad. I had to move from the 21mg early cause it was just too much nicotine!
nrt_skinirritation	States NRT patch causes skin irritation	Anybody else's skin being irritated by the patches? I get itchy from mine too.
nrt_stickissue	States NRT patch won't stick	I know that with sweat the patches don't stay on I have problems with mine everyday. My patches won't stay on.
off-topic	Discusses other topic, eg, small talk, weather, news	Now i gotta go check mine!!! I skipped breakfast and went right for lunch.
quitdate	States something about quit date	Hey my quitDate is today. My last pack of smokes. Friday is the day!
savingmoney	States money they spend per pack or how much they save by quitting	I'm putting away the money I save into an account and then can do something fun! All the money spent to damage health.
scared	States being scared/nervous/anxious/fearful about quitting or sometimes NRT	I am really nervous about quitting but ready. I've not tried the gum yet. I'm scared of it lol.
smokefree	States success in being smoke-free	I'm still smokefree starting week 2. YAY. I made it thru without smoking. Starting Day 2. Yeah.
smokingless	Has not yet quit, only able to cut down on cigs smoked OR starting to quit but still smokes some	Can’t say I've completely quit. But 4 or 5 a day is less than 15. Proud moment: only 1 cigarette today. Never thought I'd get this far.
stress	States feeling stress	I think stress has been tough but I'm not giving up. Last week work was really stressful had a lot going on at once.
support	Supports other participants	Good for you for not giving up. You got this!!! You're doing great! wooohooo!!!
tiredness	States feeling tired due to unspecified reasons or quitting smoking, not NRT	I have been so tired lately. I'm wondering if it is connected to quitting smoking. So very tired...
weightgain	States gaining weight or eating more or anything about weight or exercise	Ever since I quit I eat more so I'm going to start eating healthier and exercise. I have gained 7 pounds!

a NRT: nicotine replacement therapy.

The annotated dataset was divided into 3 mutually exclusive and collectively exhaustive subsets: training, evaluation, and test. The test dataset consisted of messages from support groups 30 to 36. From the remaining groups out of the total 45, 75% of the messages were used for training and 25% for evaluation. The dataset split was stratified based on the distribution of intent categories, independent of group membership. The training dataset was used to fine-tune the large language model, allowing it to learn patterns and features from the annotated examples. During training, the model iterated over the dataset multiple times (epochs), improving its performance by minimizing errors. The evaluation (validation) dataset was used to assess the performance of different versions of the large language model based on its training. Since it was unseen by the model during training, it served as an unbiased measure to select the best-performing model versions. The test dataset was reserved for reporting the final classification results. Also unseen by the model, it was used to evaluate the generalizability and robustness of the trained model, providing an estimate of how well the model would perform when deployed in practical settings. Table 2 summarizes the distribution of annotated messages across the 3 datasets.

**Table 2.** Distribution of smoking-cessation message intents across the training, validation, and test datasets used in this intent detection study (United States, 2012‐2014 and 2016‐2019).

Intent category	Train dataset (n=46,647)	Evaluation dataset (n=15,550)	Test dataset (n=15,219)
cigsmell	328	109	76
cravings	1446	482	267
ecigs	205	68	73
fail	774	258	152
greetings	1828	610	490
health	847	283	265
nrt_dontwork	307	102	72
nrt_dreams	258	86	54
nrt_howtouse	354	118	105
nrt_itworks	615	205	135
nrt_mouthirritation	140	46	21
nrt_nauseous	122	41	23
nrt_od	81	27	21
nrt_skinirritation	155	51	35
nrt_stickissue	188	63	83
off-topic	26,093	8698	9664
quitdate	1279	427	318
savingmoney	455	152	90
scared	293	98	83
smokefree	3072	1024	818
smokingless	285	95	33
stress	508	169	148
support	6330	2110	1983
tiredness	153	51	43
weightgain	531	177	167

### Ethical Considerations

The messages annotated in this study came from two mobile health, smoking-cessation intervention trials, which were supported by U.S. National Institutes of Health grants R34 and R01. The R34 (conducted 2012‐2014) received human subjects (HS) approval through the University of California, Irvine (UCI) institutional review board (IRB) HS# 2010‐7990 and the University of California (UC) Reliance 261: “Twitter-Enabled Mobile Messaging for Smoking Relapse Prevention.” The R01 (conducted 2016‐2019) received human subjects approval through UCI IRB HS# 2014‐1303 and UC Reliance 977: “Twitter-Enabled Mobile Messaging for Smoking Relapse Prevention (Phase 2).” Participants were compensated for enrolling with 8 weeks of NRT (approximate value US $200) and up to US $110 for survey completions. For this secondary data study, messages were analyzed verbatim after removing all identifying information, such as usernames. Participants provided informed consent for the primary data collection, and the UCI IRB allows for secondary data analysis of deidentified data without additional consent.

### Large Language Model Without Fine-Tuning

The first stage of our methodology was classifying message intents using a large language model without any fine-tuning. In other words, the model was tasked with classifying text into the observed message intent categories without any exposure to messages annotated with these intent categories in a training dataset. As a common example, a large language model might be prompted to classify the intent of a message stating, “I am very sad” as either positive or negative in sentiment, and it might correctly identify the intent as negative, despite not having been trained on the specific dataset. Since large language models are trained on vast and diverse text corpora, they have acquired an understanding of syntax, semantics, grammar, and sentiment, making them well-suited for general-purpose language tasks such as intent detection, sentiment analysis, and topic summarization.

When a large language model is used for a classification task without fine-tuning, it is guided through a prompt to emulate the task. For example, in the classification task example above, a suitable prompt might be “Classify the sentiment of the following text into one of the two categories {positive, negative}: I am very sad,” which is presented to the large language model. Then the model uses its implicit learning and reasoning to perform the task. The model works by predicting the most probable next word in the sequence. For instance, in this example, the language model may evaluate the prompt by considering potential sentiment categories such as positive and negative. Drawing on its prior knowledge and contextual understanding, it may assign a higher probability to negative based on the input text and subsequently predict negative as the final classification. The main advantage of this approach is that it does not require annotation of message intents. The only requirement is to provide the test dataset to the large language model, which then performs the classification based solely on its prior knowledge. This approach saves time and computation resources.

However, the concern is that the domain-specific task may involve text that is not part of the dataset used for pretraining the model. For example, in the domain of smoking cessation, the intent behind the messages can often be highly domain-specific as health-intervention communications tend to be confidential. A message such as “I used gum today and wanted to smoke two hours later,” might be interpreted by a language model as indicating a cessation failure. However, within the context of our smoking-cessation dataset, this message corresponds to a more specific intent, nrt_dontwork, which signifies that the user perceived NRT as ineffective.

Thus, knowledge of the domain becomes crucial for getting higher accuracy with large language models. When a model lacks domain-specific knowledge, its performance on related tasks tends to decline. Moreover, in the absence of domain-specific knowledge, large language models may introduce biases or errors stemming from the datasets on which they were originally trained, possibly causing hallucinations. Furthermore, large language models without domain knowledge tend to struggle with classification tasks involving numerous similar intent categories, such as our categories of nrt_dontwork and nrt_itworks, which refer to NRT being ineffective or effective, respectively. While these intents have completely different meanings, large language models that lack fine-tuning often cannot distinguish between them.

In this study, we assessed the performance of a large language model without fine-tuning at completing the task we required for our mobile health application: recognizing the 25 main intents of messages related to smoking cessation, which included numerous nuanced messages about using NRT. We used the state-of-the-art but off-the-shelf Llama-3 8B by Meta. We supplied this model with descriptions of all 25 of our intent categories, as defined in Table 1, to facilitate its classification process and ensure that its predictions were constrained to these predefined intent categories.

### Large Language Model With Fine-Tuning

The next stage of our methodology was fine-tuning the same large language model, Llama-3 8B, using our domain-specific dataset with smoking-cessation messages. Fine-tuning a large language model refers to further training the model on one or more datasets from the targeted domain, the goal being to transform a general-purpose model into one specialized for the domain for improved performance. Domain-specific knowledge is often crucial for large language models because the datasets used in the initial basic training often lack the detailed context relevant to the specific task. Fine-tuning enables the models to learn domain-relevant nuances, semantics, and reasoning.

For example, during fine-tuning, the model might be exposed to messages about NRT gum aiding or not aiding with smoking cessation, for instance, “I used gum today and wanted to smoke two hours later.” Fine-tuning allows the model to learn subtle distinctions and accurately differentiate between semantically similar message intents. For instance, a model without fine-tuning might incorrectly predict the message intent here as fail (failure at smoking cessation). However, with fine-tuning on the domain-specific dataset, the model should learn that the user is talking about their NRT gum failing to help them with nicotine cravings, and therefore the correct intent is nrt_dontwork.

On the other hand, fully training a large language model on a domain-specific dataset risks the model forgetting its previously acquired general knowledge. Consequently, the model might forget critical knowledge related to language comprehension, reasoning, and generalizing across tasks. Therefore, instead of completely retraining the model, we undertake fine-tuning because it preserves and often freezes the original model weights (ie, the models’ prior knowledge) while learning new weights (new knowledge) that is built upon the existing foundation. So, this approach enables the model to retain its previously acquired knowledge while simultaneously incorporating new insights from the domain-specific dataset.

Therefore, we fine-tune the Llama-3 8B large language model on our smoking-cessation dataset. We selected Llama-3 8B to balance computational efficiency and model performance with feasibility for in-house fine-tuning and inference, considering our resource constraints. Meta’s Llama-3 8B, when used in causal mode, is designed to predict the next word in a sequence sequentially. When used in this mode, intent detection requires specialized prompting, for example, “Input: I am feeling very sleepy. So the intent of this message is:.” This prompt is sent to the model which then predicts the next probable word as tiredness. This prompt can be formulated in various ways; however, the approach has several limitations. It is highly dependent on the prompt design and it operates indirectly through generation, which reduces efficiency, makes end-to-end training more challenging, and necessitates careful prompt engineering and output parsing. Often, the model’s output may fall outside the predefined intent categories; for instance, in the previous example where the main intent is tiredness, the model might generate a related but nonfocal intent such as drained.

For classifying message intents, we used a version of the Llama-3 model that is specifically adapted for classification rather than using the standard causal mode designed for language generation. Instead of predicting the next word in a sentence, this version of the model learns to assign entire messages to intent categories. To achieve this, we added a lightweight classification layer which works as the category chooser producing the final model results. This layer enables the model to directly select the most appropriate category for each input message. Importantly, this approach allows us to begin to address the issue of dataset imbalance, where some intent categories are more common than others. By assigning more weight to underrepresented categories during fine-tuning, the model becomes more attentive to less frequent but clinically important message types.

**Figure 2.**
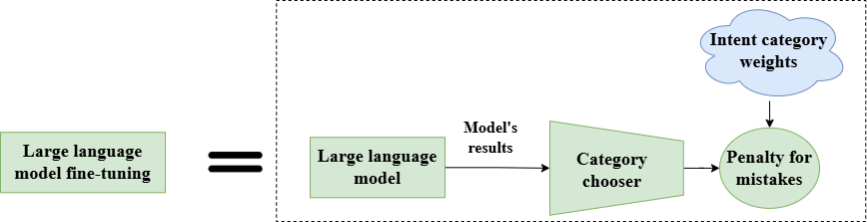
Large language model fine-tuning used in this intent detection study (United States).

Additionally, this approach gives us a probability score for each category, which helps us understand how confident the model is in its predictions. This is valuable for downstream decision-making and human oversight. Figure 2 provides an overview of this fine-tuning process, showing that the model’s outputs feed into the category chooser, which then predicts the most appropriate intent category. During fine-tuning, we assign more importance to underrepresented categories when the model makes mistakes. This is done by adding category weights to the penalty for mistakes, helping the model pay more attention to rare but clinically important intent categories. We fine-tune the Llama-3 8B model in this way to improve its ability to detect nuanced user intents in smoking-cessation conversations, for example, related to the use of the free NRT provided as a cessation aid.

### Large Language Model With Fine-Tuning and Downsampling

The third stage of our methodology was downsampling the dominant off-topic (domain-unrelated) message intent category and then fine-tuning the model on the updated dataset. Dataset imbalance is a typical problem in classification tasks, meaning that the number of instances (samples or examples) often varies quite substantially across the different focal categories. For example, in our dataset, the off-topic intent category contained 44,445 samples, whereas the nrt_od category contained only 129 samples. In a multicategory classification task, if a particular category has a much higher number of samples than the other categories, the model being trained tends to be biased toward choosing the category with the higher number of samples. In other words, in the training dataset, the model sees many more samples of this majority category (eg, off-topic) and ends up favoring it in its predictions. Thus, the minority categories (eg, nrt_od) are underpredicted.

For our application which is to accurately predict message intents in smoking-cessation support groups, both majority and minority intent categories require accurate predictions. But, if we train the model with all our data, the model will tend to be more accurate in predicting the highly dominant off-topic category and less accurate in predicting the minority categories with fewer samples. Additionally, the off-topic intent category refers to messages considered irrelevant or uninformative for smoking cessation or support group bonding; it falls outside the 24 key intent categories. Knowing this gives us even more leeway to address our severe category imbalance by reducing the volume of off-topic category examples. Doing this will not compromise the data from the other 24 intent categories.

So for our analysis, we have downsampled the off-topic intent category. Downsampling is a method used to mitigate category imbalance within a dataset by decreasing the sample size of the majority category or categories to better align with sample sizes of the minority categories. We downsampled the instances of the off-topic category to mirror the average number of instances across the other 24 categories. We consistently applied this approach to our training, test, and evaluation datasets. To determine the appropriate level of downsampling for the large off-topic category, we experimented with several approaches, evaluated them on the evaluation dataset, and selected the best-performing approach, which was the average instances across all other category data. These results are reported in Multimedia Appendix 1. After this, we fine-tuned the model using the same approach as in the Large Language Model With Fine-Tuning section. After downsampling the off-topic intent category, the model was fine-tuned using category weights to penalize misclassification of underrepresented categories, ensuring they received sufficient attention during fine-tuning. Although downsampling reduced the disproportionality of the off-topic category, the dataset remained imbalanced, and further downsampling of clinically meaningful categories was not desirable due to the risk of losing valuable information. Thus, category weighting was applied to enhance learning of the rare but important categories while maintaining balance in our fine-tuning process.

The typical limitations of downsampling include loss of information, reduced generalizability, and degraded intent-detection performance in the dominant category. However, these concerns were less applicable in our setting, as we only downsampled the off-topic intent category. This category is unrelated to the 24 focal intent categories and largely represents noise, so reducing its representation is less problematic. In other words, failing to detect some off-topic messages is less harmful than misclassifying important on-topic messages. Even if the detection of off-topic intents decreases, as long as the 24 on-topic intents are accurately recognized, the system remains effective. Misclassifying an off-topic message intent as 1 of the 24 focal intents keeps the chatbot response within the smoking-cessation domain, which is desirable.

### Large Language Model With Fine-Tuning, Downsampling, and Error Correction

The final stage in our methodology was downsampling the dominant off-topic intent category, fine-tuning the model, correcting human annotation errors flagged by examining model-human disagreements in the intent classifications, and fine-tuning the model again. Since the datasets were manually annotated, they were susceptible to human annotation errors. Though large language models are powerful tools due to their training on extensive datasets, fine-tuning them on imperfectly annotated or noisy data can negatively impact their learning and classification performance.

Model-human disagreement on a message’s intent category can reveal a weakness in either the model or the human annotation. The model could have incorrectly classified the user’s intent, the human annotators could have done so, or possibly both. Therefore, identifying model-human disagreement over how to classify message intents and resolving the human-caused annotation errors enables us to improve the final dataset we use for model fine-tuning. If the model and humans disagree, reviewing these disagreements helps us identify human annotation mistakes, for example, due to messages being ambiguous or vague, or general human oversights in annotations. The human annotation errors can then be corrected in the dataset, and fine-tuning can proceed with less noisy data.

Thus, we analyzed model-human disagreements across the downsampled training, evaluation, and test datasets to identify potential human annotation errors. A domain expert independently reviewed each flagged case and corrected original annotations only when the original human one was incorrect; the model’s prediction was never adopted as the new annotation. Only the corrected training dataset was used for fine-tuning again along with the corrected evaluation dataset, and the corrected test dataset remained a true holdout throughout this process. The final performance scores were computed on the expert-validated test dataset, ensuring that the scores remained unbiased and free of data leakage. This approach also ensured consistency across training, evaluation, and testing, as all datasets were corrected using the same procedure and verified by the same domain expert. Category weighting was also applied in this method to enhance the model’s learning of rare but important categories while ensuring a balanced fine-tuning process.

### Fine-Tuning Hyperparameters

For reproducibility, we provide details on our fine-tuning and inference setup here. We fine-tuned the Llama-3 8B model for 25-category sequence classification using 4-bit quantized low-rank adaptation (QLoRA) within the parameter-efficient fine-tuning (PEFT) framework. Low-rank adaptation (LoRA) adapters (rank=64, *α*=16) were applied to the query projection (q_proj), key projection (k_proj), value projection (v_proj), and output projection (o_proj) layers, with a dropout rate of 0.05. The category chooser (classification head) is a single linear layer (fully connected) that maps the large language model’s hidden dimension of 4096 to the 25 intent categories. Fine-tuning was performed in 32-bit floating point (FP32) precision with 4-bit quantization for memory efficiency along with the Adaptive Moment Estimation With Weight Decay (AdamW) optimizer with a learning rate of 1×10^–^⁴, weight decay of 0.01, batch size of 8, and 5 epochs. Early stopping with a patience of 7 monitored unweighted average precision was used to load the best model. Category (class) weights were set inversely proportional to label frequency (normalized) to address category imbalance. Fixed random seeds (42, 123, and 2023) were used for both CPU (central processing unit) and GPU (graphics processing unit), with deterministic Compute Unified Device Architecture (CUDA) operations for reproducibility on the NVIDIA GeForce RTX 4090 GPU. Tokenization used a maximum sequence length of 512, padded to the model’s end-of-sequence (EOS) token. Evaluation and checkpoint saving occurred every 1000 steps. During inference, computation was performed using Brain Floating Point 16 (bfloat16) precision with 4-bit quantization for memory efficiency.

## Results

### Evaluation Metrics

To evaluate the different large language model training approaches for recognizing message intents, we used 3 standard accuracy measures:

Precision: How often the model was correct when it predicted a certain intent.

$Precision=\frac{Correct\;predictions\;for\;an\;intent}{Total\;predictions\;made\;for\;that\;intent}$

Recall: How often the model successfully recognized an intent when a message conveyed it.

$Recall=\frac{Correct\;predictions\;for\;an\;intent\;}{Total\;actual\;cases\;of\;that\;intent}$

*F*_1_-score: A numeric combination of precision and recall.

$F_{1}-score=2\times \frac{Precision\;\times \;Recall}{Precision\;+\;Recall}$

### Aggregate Performance

**Figure 3.**
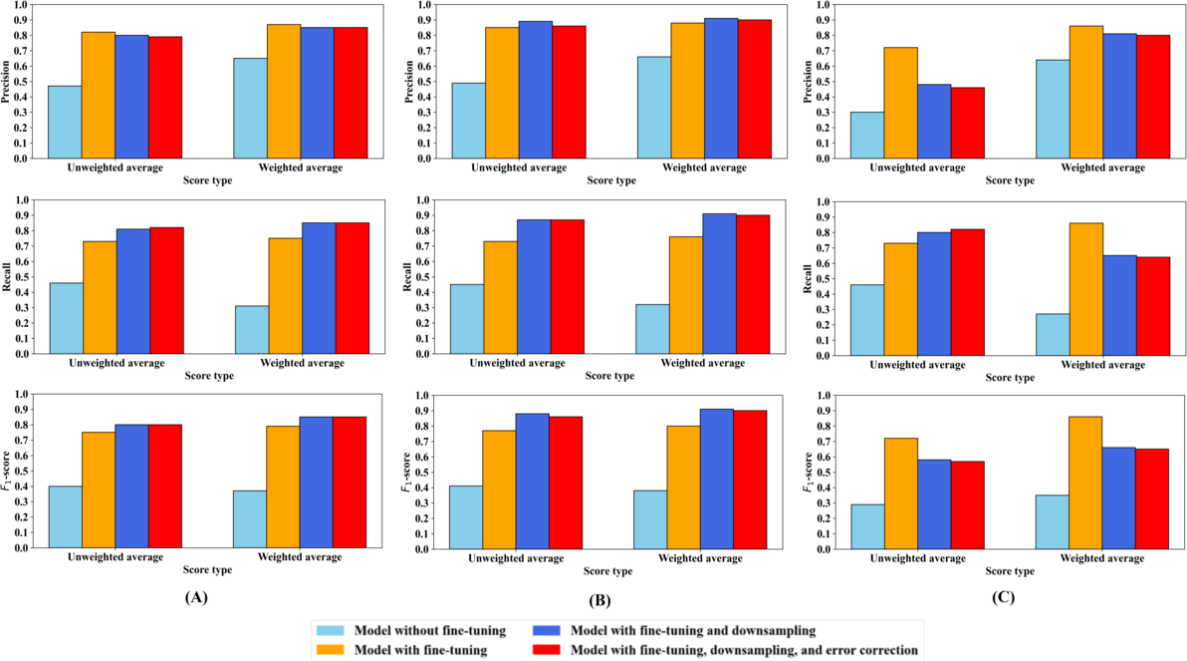
Overall unweighted and weighted precision, recall, and *F*_1_-scores for 4 large language model training methods for intent detection in smoking-cessation messages in this study (United States). Methods were evaluated on the (A) downsampled uncorrected test dataset, (B) downsampled corrected test dataset, and (C) full test dataset.

Figure 3 summarizes the performance of the Llama-3 8B model across the 4 training methods. The downsampling-only and downsampling with error correction methods involved modifying the training dataset through reduction of off-topic intent samples and annotation error correction, respectively, so we evaluated all 4 training methods using 3 distinct test datasets. Evaluation results are shown in Figure 3A using the downsampled uncorrected test dataset, in Figure 3B using the downsampled corrected test dataset, and in Figure 3C using the full unmodified test dataset.

In Figure 3A, which uses the downsampled uncorrected test dataset, the off-the-shelf model with extensive pretraining but no domain-specific fine-tuning performed poorly at recognizing message intents. It achieved unweighted *F*_1_-score, precision, and recall of 0.40, 0.47, and 0.46, respectively, and weighted *F*_1_-score, precision, and recall of 0.37, 0.65, and 0.31, respectively. The unweighted scores assigned equal importance to each intent category, while the weighted scores gave greater weight to categories with more instances. The low scores highlight the complexity of the classification task, demonstrating that intent classification in the mobile health context studied was not straightforward and required specialized fine-tuning to achieve adequate performance. The domain-specific fine-tuning method achieved a much-improved unweighted *F*_1_-score, precision, and recall of 0.75, 0.82, and 0.73, respectively, and weighted *F*_1_-score, precision, and recall of 0.79, 0.87, and 0.75, respectively. The method of fine-tuning after downsampling the dominant off-topic intent category improved the unweighted *F*_1_-scores from 0.75 to 0.80 and yielded unweighted precision and recall of 0.80 and 0.81, respectively. The weighted *F*_1_-score, precision, and recall were all about 0.85. The fine-tuning, downsampling, and error correction method performed comparably to the downsampling-only method, achieved unweighted *F*_1_-score, precision, and recall of 0.80, 0.79, and 0.82, respectively, and similar weighted scores.

In Figure 3B, which uses the downsampled corrected test dataset, the method with no fine-tuning obtained an unweighted *F*_1_-score, precision, and recall of 0.41, 0.49, and 0.45, respectively, and a weighted *F*_1_-score, precision, and recall of 0.38, 0.66, and 0.32, respectively. Fine-tuning yielded an unweighted *F*_1_-score, precision, and recall of 0.77, 0.85, and 0.73, respectively, and a weighted *F*_1_-score, precision, and recall of 0.80, 0.88, and 0.76, respectively. The relatively lower recall indicates the model still struggles to consistently identify certain intents when present. The downsampling and fine-tuning method achieved an unweighted *F*_1_-score, precision, and recall of 0.88, 0.89, and 0.87, respectively, and a weighted *F*_1_-score, precision, and recall of 0.91 for all three. The method of downsampling, fine-tuning, and error correction achieved an unweighted *F*_1_-score, precision, and recall of 0.86, 0.86, and 0.87, respectively, and a weighted *F*_1_-score, precision, and recall of 0.90 for all three. Although error correction yielded good performance, the downsampling-only approach achieved slightly higher unweighted precision and higher weighted scores.

In Figure 3C, which uses the full test dataset, the method without fine-tuning achieved an unweighted *F*_1_-score, precision, and recall of 0.29, 0.30, and 0.46, respectively, and a weighted *F*_1_-score, precision, and recall of 0.35, 0.64, and 0.27, respectively. Among all test cases, 991 predictions could not be matched to any of the 25 intent categories (eg, the model predicted “stick” instead of “stickissues”); these samples were included in the final scoring as misclassifications. The fine-tuning method achieved an unweighted *F*_1_-score, precision, and recall of 0.72, 0.72, and 0.73, respectively, and a weighted *F*_1_-score, precision, and recall of 0.86 for all three. The fine-tuning and downsampling method attained an unweighted *F*_1_-score, precision, and recall of 0.58, 0.48, and 0.80, respectively, and a weighted *F*_1_-score, recall, and precision of 0.66, 0.81, and 0.65, respectively. The final method that added error correction achieved an unweighted *F*_1_-score, precision, and recall of 0.57, 0.46, and 0.82, respectively, and a weighted *F*_1_-score, precision, and recall of 0.65, 0.80, and 0.64, respectively. Although both methods demonstrated comparable overall performance, the error correction method yielded a higher unweighted recall, indicating its robust performance at detecting intents in real-world, noisy datasets. Using the full uncorrected test dataset lowered the scores for our last two methods due to the large number of off-topic intents in the test dataset, which had been downsampled in the training dataset. This led to many off-topic intents being wrongly predicted as in-domain intents; however, this was acceptable to us as the main goal was to accurately identify the 24 in-domain intents. Misclassifying off-topic intents as in-domain allowed for in-domain chatbot responses focused on the smoking-cessation domain. Moreover, even when we used the full test dataset, our last two methods sequentially improved recall. These improvements suggest that our last training steps helped the model learn to identify the relevant in-domain intents more effectively in the noisy real-world data, which was our main goal. In other words, unweighted recall served as the more informative evaluation metric for our application.

In Figures 3A and 3B, the differences between weighted and unweighted scores were relatively small because of the downsampled off-topic intent category. However, in Figure 3C, the discrepancy was more pronounced because the full test dataset contained a large number of off-topic intent samples, and in the weighted scores, each intent category’s contribution is proportional to its sample size, so more frequent intents are treated as more important. In our application, the unweighted recall scores are particularly important, as these give equal importance to all intents regardless of their frequency.

To assess result consistency, we fine-tuned our final method of downsampling with error correction using three random seeds (42, 123, and 2023) to account for variability in model initialization. We observed a mean unweighted *F*_1_-score of 0.85 (SD 0.01) and a mean weighted *F*_1_-score of 0.89 (SD 0.01) on the downsampled corrected dataset, demonstrating high consistency. Additionally, for our last two methods, we used bootstrap resampling, a statistical method that repeatedly evaluates the model on slightly different versions of the test dataset to estimate performance variability. For the downsampling and error correction method, the unweighted *F*_1_-scores ranged from 0.85 to 0.88 with 95% confidence. For the downsampling-only method, the unweighted *F*_1_-scores ranged from 0.86 to 0.89 with 95% confidence. Both results indicate stable performance.

We conducted a bootstrap resampling analysis with replacement (1,000 resamples; random seed=42) on the full test dataset and computed the unweighted-average recall for the downsampling-only and the downsampling and error correction methods. A paired 2-tailed *t* test on the bootstrap differences indicated that error correction significantly improved unweighted-average recall (*t*_999_=69.5, *P*<.001). The 95% CI for the improvement was 0.0022-0.0292, suggesting a small but consistent gain over downsampling alone.

### Performance by Message Intent Category

#### Precision for Each Intent Category

**Figure 4.**
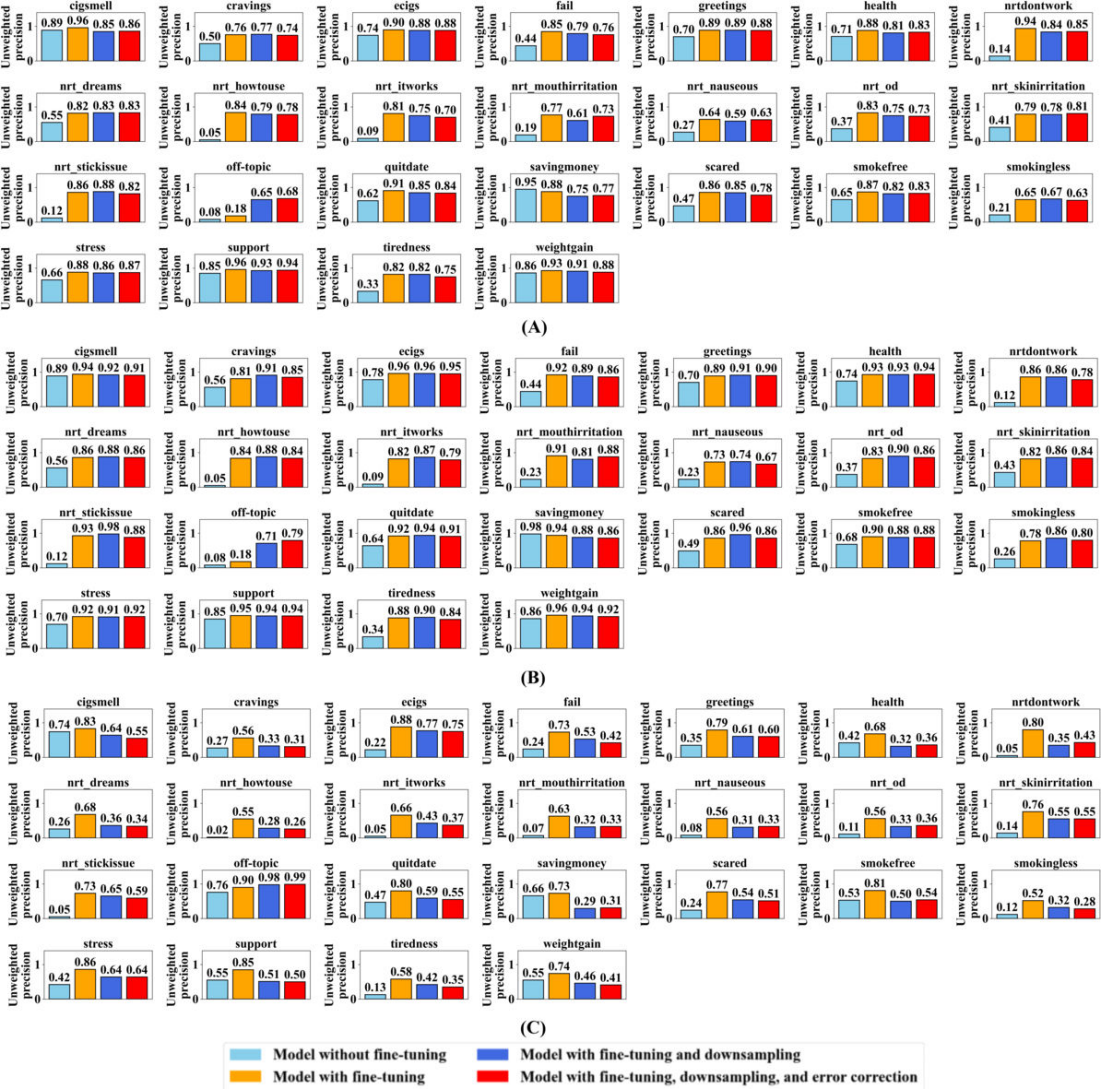
Unweighted precision scores for the 4 large language model training methods used to detect intents in smoking-cessation messages in this study (United States). Methods were evaluated on the (A) downsampled uncorrected test dataset, (B) downsampled corrected test dataset, and (C) full test dataset.

Figure 4 presents the unweighted precision scores for predicting each of the 25 intents across the different training methods. Unweighted precision reflects the proportion of correct predictions among all instances predicted for a given intent and requires balanced performance across intents. Evaluation results are shown in Figure 4A using the downsampled uncorrected test dataset, in Figure 4B using the downsampled corrected test dataset, and in Figure 4C using the full unmodified test dataset.

In Figure 4A, using the downsampled uncorrected test dataset, for the method without fine-tuning, the precision for most intents was around 0.47, with a few categories slightly exceeding that score but many falling below it, indicating weak overall performance. With fine-tuning, the precision for most intents improved to around 0.82. However, the dominant off-topic intent achieved a low precision of 0.18, reflecting a classification bias. Its large number of training samples caused the model to overpredict it, reducing precision. In effect, other important intents were often misclassified as off-topic, which is concerning because this can suppress the detection of key intents. The method of fine-tuning and downsampling of the dominant off-topic intent yielded close to a 0.80 precision score for most intents, but certain intents fell below the 0.80 mark. The final approach, combining fine-tuning, downsampling, and error correction, achieved precision comparable to the downsampling-only method.

In Figure 4B, using the downsampled corrected test dataset, the method without fine-tuning exhibited a precision of approximately 0.49 across most intents. With fine-tuning, precision improved to around 0.85 for most intents, although the off-topic intent category continued to show a low precision of 0.18. Fine-tuning with downsampling resulted in many precision scores close to 0.89. Adding error correction yielded an average precision of 0.86. Thus, the downsampling-only method demonstrated more robust performance on precision.

In Figure 4C, using the full dataset, the method without fine-tuning exhibited a precision of approximately 0.30 for most intents. With fine-tuning, precision improved to around 0.72 for most intents, although the dominant off-topic intent achieved a higher precision of 0.90. Fine-tuning with downsampling resulted in a precision mainly close to 0.48. The final method of combining fine-tuning, downsampling, and error correction achieved an average precision of 0.46. The large volume of off-topic intent samples in the full test dataset led to many off-topic intents being misclassified as in-domain intents, lowering the precision scores for the last two methods.

#### Recall for Each Intent Category

**Figure 5.**
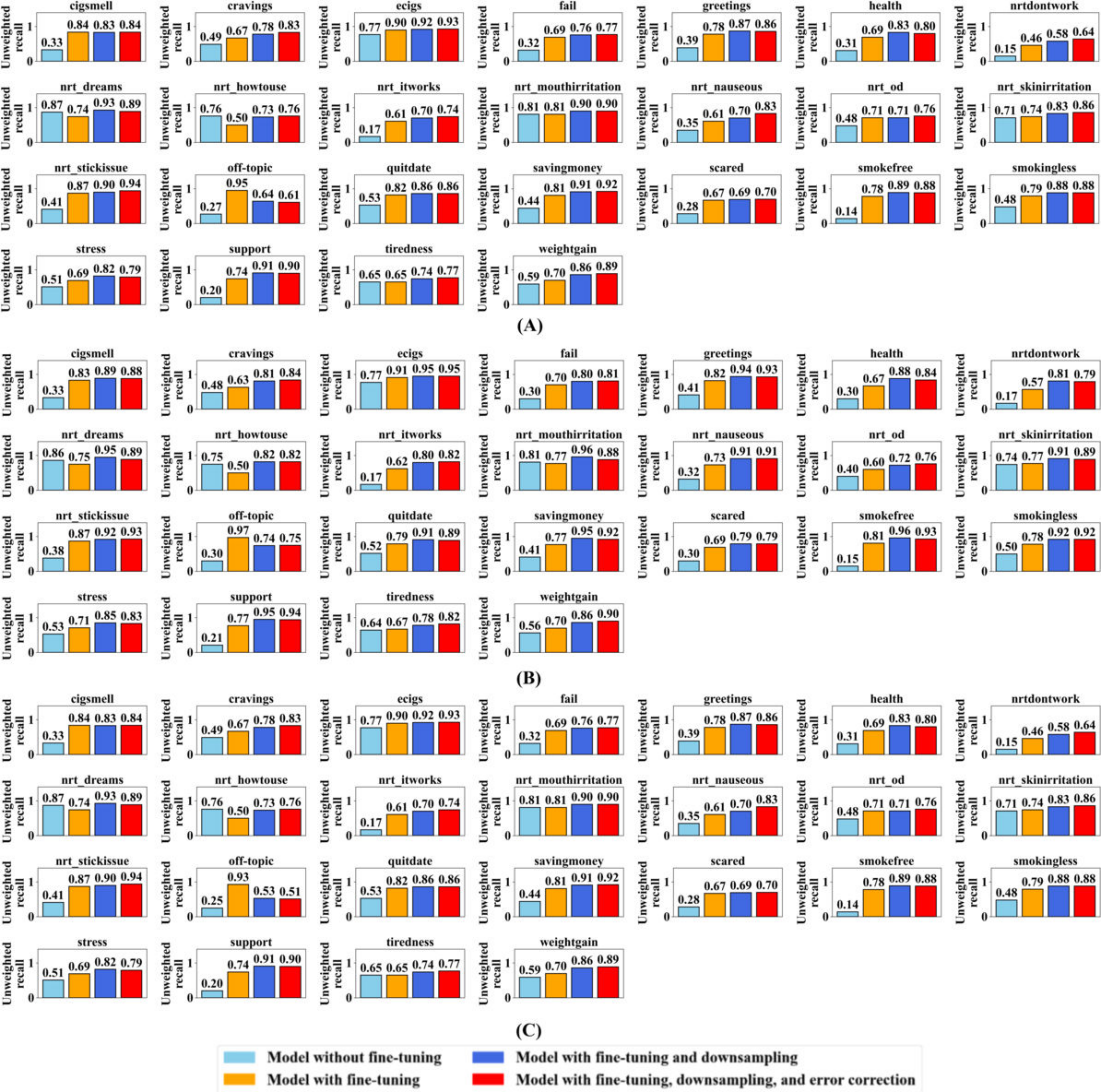
Unweighted recall scores for the 4 large language model training methods used to detect intents in smoking-cessation messages in this study (United States). Models were evaluated on the (A) downsampled uncorrected test dataset, (B) downsampled corrected test dataset, and (C) full test dataset.

Figure 5 presents the unweighted recall scores for each of the 25 intents across the different training methods. Unweighted recall shows how accurately the model picks the actual intents and requires balanced performance across intents. Evaluation results are shown in Figure 5A using the downsampled uncorrected test dataset, in Figure 5B using the downsampled corrected test dataset, and in Figure 5C using the full unmodified test dataset.

In Figure 5A, using the downsampled uncorrected test dataset, the method without fine-tuning achieved a recall of approximately 0.46 for most intents, with some slightly higher but many lower. Fine-tuning improved recall to around 0.73 for most intents, although several still fell below this, while the dominant off-topic intent category reached 0.95 recall. Adding downsampling, many intents achieved a recall close to 0.81, though some remained lower. Adding error correction as well produced slightly better recall across several intents, averaging 0.82, indicating strong overall performance.

In Figure 5B, using the downsampled corrected test dataset, the method without fine-tuning achieved a recall of approximately 0.45 for most intents. With fine-tuning, recall improved to around 0.73 for most intents, while the dominant off-topic intent category reached 0.97. Adding downsampling, many intents achieved recall close to 0.87, though some remained lower. Adding error correction as well achieved an average recall of 0.87 across intents.

In Figure 5C, using the full test dataset, the method without fine-tuning achieved a recall of approximately 0.46 for most intents. With fine-tuning, recall improved to around 0.73 for most intents, while the dominant off-topic intent category reached 0.93. Adding downsampling, many intents achieved a recall close to 0.80. Adding error correction as well achieved the highest average recall of 0.82 across intents, demonstrating strong overall performance on noisy, real-world data.

#### *F*_1_-Scores for Each Intent Category

**Figure 6.**
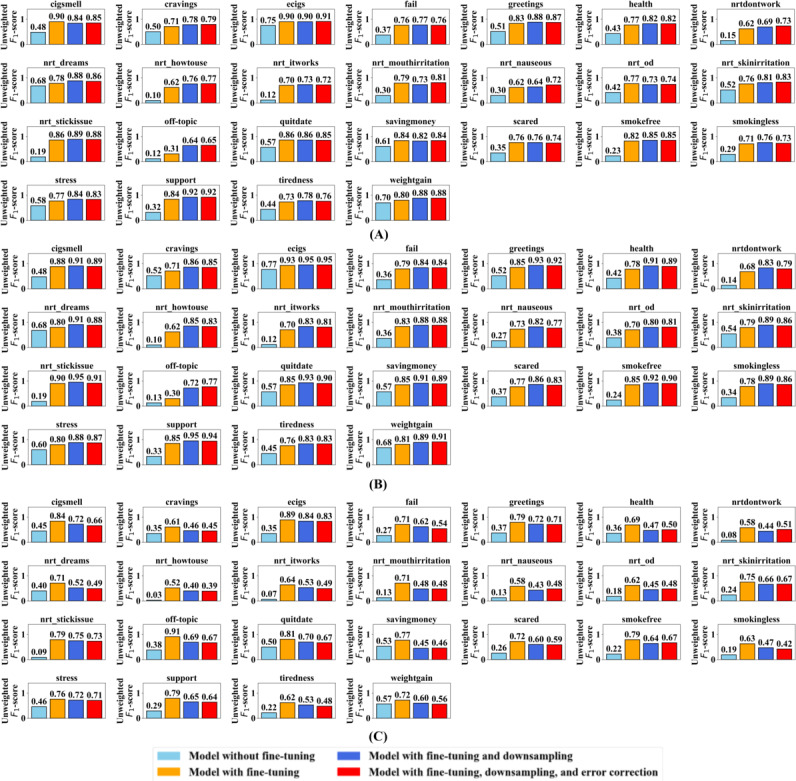
Unweighted *F*_1_-scores for the 4 large language model training methods used to detect intents in smoking-cessation messages in this study (United States). Models were evaluated on the (A) downsampled uncorrected test dataset, (B) downsampled corrected test dataset, and (C) full test dataset.

Figure 6 presents the unweighted *F*_1_-scores for each of the 25 intents across the different training methods. Unweighted *F*_1_-score combines precision and recall and emphasizes balanced performance across intent categories. Evaluation results are shown in Figure 6A using the downsampled uncorrected test dataset, in Figure 6B using the downsampled corrected test dataset, and in Figure 6C using the full unmodified test dataset.

In Figure 6A, using the downsampled uncorrected test dataset, the model without fine-tuning achieved an *F*_1_-score of approximately 0.40 across most intents, although several intents scored lower. Fine-tuning increased the *F*_1_-score to about 0.75, but many intents scored lower, and the off-topic intent category scored about 0.31. The downsampling method with or without error correction achieved an *F*_1_-score of about 0.80, though some intents were lower.

In Figure 6B, using the downsampled corrected test dataset, the model without fine-tuning achieved an *F*_1_-score of approximately 0.41 across most intents. Fine-tuning improved the *F*_1_-score to about 0.77, though many intents scored lower, and the off-topic intent category scored about 0.30. The downsampling method achieved an *F*_1_-score of about 0.88, but some intents remained lower, while adding error correction achieved *F*_1_-scores of about 0.86 across intents.

In Figure 6C, using the full test dataset, the model without fine-tuning achieved an *F*_1_-score of about 0.29 across most intents, with several lower. Fine-tuning increased the *F*_1_-score to about 0.72, though many intents scored lower, and the off-topic intent category scored above 0.91. The downsampling method yielded an *F*_1_-score of 0.58, while adding error correction yielded an *F*_1_-score of 0.57, but several intents were lower.

### Total Statistics on Model-Human Disagreement

**Figure 7.**
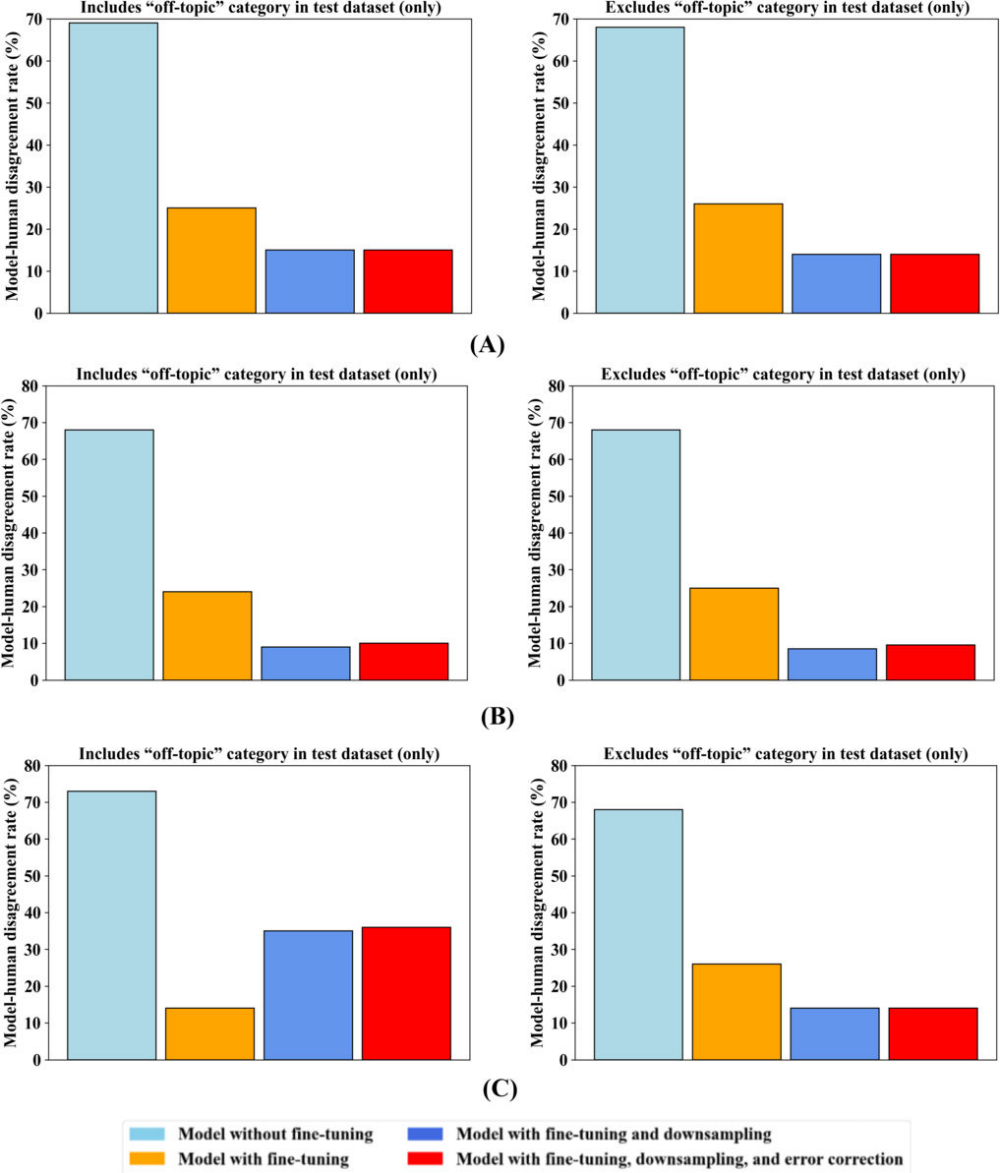
Model-human disagreement, including and excluding the dominant off-topic intent category in the test dataset, for the 4 large language model training methods studied (United States). Methods were evaluated on the (A) downsampled uncorrected test dataset, (B) downsampled corrected test dataset, and (C) full test dataset.

Figure 7 shows the model-human disagreement across our 4 training methods. Model-human disagreement provides a comprehensive view of a model’s accuracy at judging a message’s intent relative to human judges of that intent. The left side of each figure includes either the full or downsampled off-topic intents in the test dataset, while the right side excludes the off-topic intents. Results are shown in Figure 7A using the downsampled uncorrected test dataset, in Figure 7B using the downsampled corrected test dataset, and in Figure 7C using the full unmodified test dataset.

In Figure 7A, using the downsampled uncorrected test dataset, the left side includes off-topic intents in the test dataset. The method with no fine-tuning yielded about 69% model-human disagreement. Fine-tuning alone lowered this to 25%. Fine-tuning with downsampling with or without error correction lowered the disagreement to about 15%. The right side of Figure 7A excludes the off-topic intents from the test dataset. It shows the method without fine-tuning yielded approximately 68% model-human disagreement. Fine-tuning alone reduced this to 26%. Fine-tuning with downsampling with or without error correction lowered the disagreement to about 14%. The results, including or excluding the off-topic intent samples, were similar because the number of off-topic samples in the downsampled test dataset was small enough to have minimal impact.

In Figure 7B, using the downsampled corrected test dataset, the left side includes off-topic intents in the test dataset. The method with no fine-tuning showed about 68% model-human disagreement. Fine-tuning alone lowered this to 24%, fine-tuning with downsampling lowered it to 9%, and adding error correction yielded 10%. The right side of Figure 7B excludes the off-topic intents from the test dataset. The method without fine-tuning yielded 68% model-human disagreement. Fine-tuning alone lowered it to 25%, fine-tuning and downsampling lowered it to 8.5%, and adding error correction yielded 9.5%.

In Figure 7C, using the full test dataset, the left side includes off-topic intents in the test dataset. The method without fine-tuning exhibited 73% model-human disagreement. Fine-tuning alone reduced this to 14%. In contrast, fine-tuning with downsampling yielded 35%, and adding error correction yielded 36%. So, on real-world noisy data, downsampling yielded more model-human disagreement than no downsampling. This occurred because downsampling the off-topic intent category trained the model to recognize rarer but important in-domain intents. The higher model-human disagreement for the last two methods was primarily due to off-topic samples being misclassified as in-domain intents. At first glance, it may appear that fine-tuning alone performs better because it produces less model-human disagreement in the original noisy data. However, this is largely an artifact of the imbalanced dataset: both the training and test datasets contain a disproportionately large number of off-topic intent samples and, since both are similarly dominated by off-topic, the disagreement rate becomes artificially low, masking poor performance on the more important in-domain intents. The right side of Figure 7C, which excludes the off-topic intents from the test dataset, provides a different and, we believe, more accurate picture. Here, the model without fine-tuning yielded 68% model-human disagreement. Fine-tuning alone lowered this to 26%, and downsampling with or without error correction method lowered it to 14%. These results indicate that when the off-topic intents are excluded from the test dataset, the downsampling-only and the downsampling and error correction methods achieve the lowest model-human disagreement on the 24 important domain-specific intents.

### Statistics on Model-Human Disagreement

**Figure 8.**
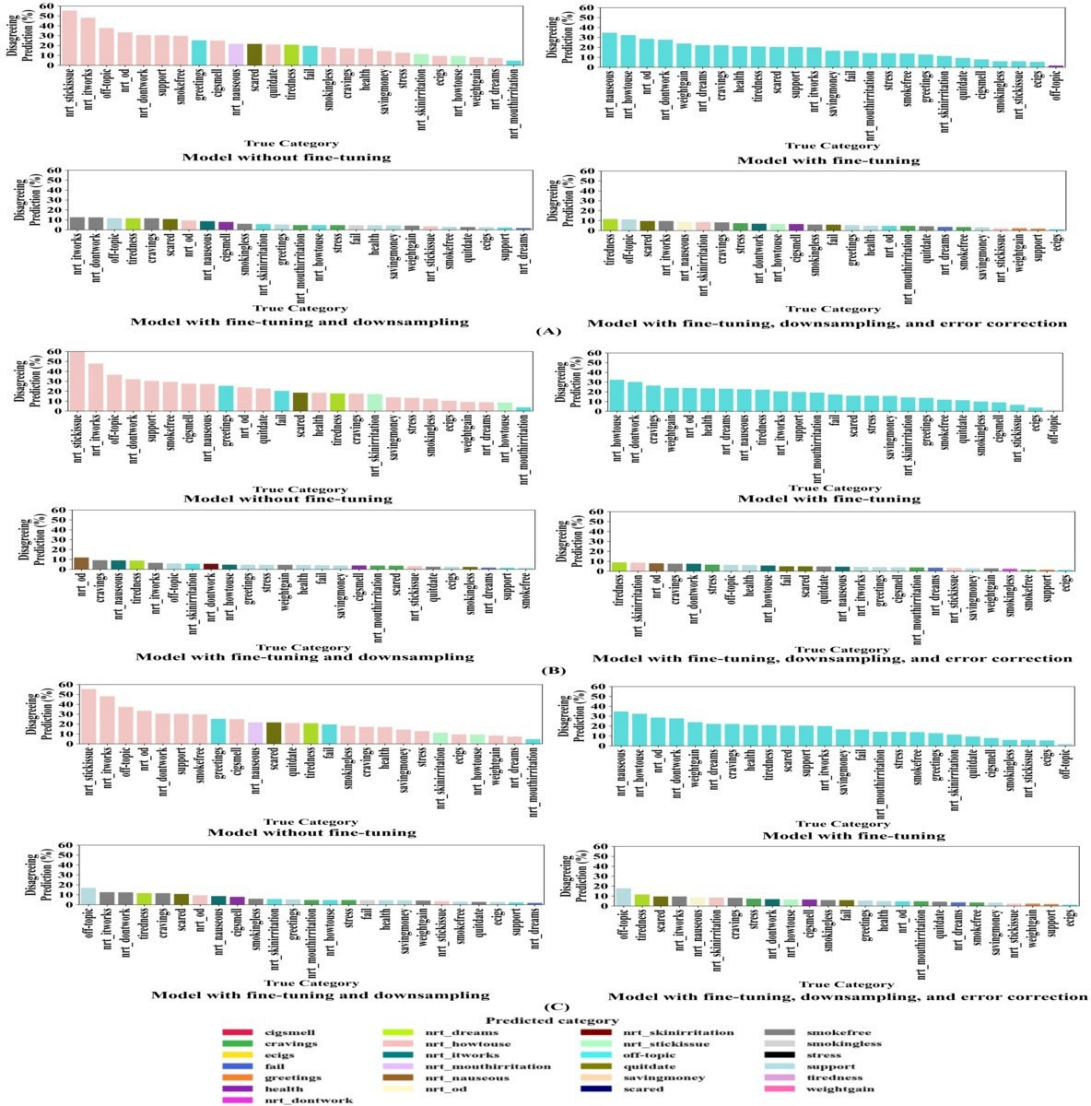
Model-human disagreement by intent category for the 4 large language model training methods studied (United States). Methods were evaluated on the (A) downsampled uncorrected test dataset, (B) downsampled corrected test dataset, and (C) full test dataset.

Figure 8 shows the percentages of model-human disagreement by intent category for our 4 training methods, which we will refer to as model mispredictions because the goal of this analysis is to understand when and how a model confused the different intent categories. This information provides insight into each training method’s specific weaknesses. In Figure 8A, evaluation was conducted using the downsampled uncorrected test dataset. In Figure 8B, evaluation was conducted using the downsampled corrected test dataset. Finally, in Figure 8C evaluation was conducted using the full unmodified test dataset.

In Figure 8A, using the downsampled uncorrected test dataset, the method without fine-tuning caused the model to mispredict the intent nrt_howtouse for many other intent categories. For some categories, over 50% of instances were mispredicted as nrt_howtouse; for 17 out of 25 categories, this incorrect prediction dominated. This demonstrates that the model without fine-tuning was heavily biased toward nrt_howtouse and failed to distinguish it from other intents. A similar issue appeared with the fine-tuned model, but the bias shifted toward the dominant off-topic intent category. Here, 24 out of 25 categories were most commonly and incorrectly mispredicted as off-topic, and this occurred approximately 30% of the time, reflecting the overrepresentation of off-topic samples. This highlights a key limitation of fine-tuning alone: without addressing category imbalance, the model struggles to predict important but underrepresented intent categories accurately. In contrast, the fine-tuning with the downsampling method produced a more balanced pattern of mispredictions. No single intent category was mispredicted, as evidenced by the absence of any overwhelmingly frequent color in our bar graph; only minor weaknesses remained. A similar pattern was observed for the fine-tuning, downsampling, and error correction method, with no single intent category mispredicted. These results demonstrate that downsampling effectively reduced bias toward mispredicting dominant intent categories and promoted more equitable performance.

In Figure 8B, using the downsampled corrected test dataset, the methods without and with fine-tuning alone showed similar patterns, with nrt_howtouse and off-topic intents being most frequently mispredicted, respectively. In comparison, the methods adding downsampling alone, or downsampling with error correction, showed a more balanced pattern of mispredictions, with no single intent category dominating, the only exception being the support intent category, with 7 different intents frequently misclassified as support, although in each case less than 5% of the time.

In Figure 8C, using the full test dataset, the methods without and with fine-tuning alone showed similar patterns, with nrt_howtouse and off-topic intents most frequently mispredicted, respectively. Adding downsampling and then also adding error correction produced a more balanced pattern of mispredictions, except for the support intent, which was mispredicted as other categories. Examples in which the model mispredicted other categories as support include: “Those are great reasons,” which was human-annotated as off-topic, “Nicotine addiction is like any other drug out there and the side effects are bad. Sorry to say,” which was human-annotated as health, and “Patches will help greatly. You can do it,” which was human-annotated as nrt_itworks. These results show that, in certain misclassification cases, the model produced a generic smoking-cessation support response rather than an intent-specific one. While such responses may provide baseline assistance, they do not substitute for the intended goal of accurate intent-specific classification.

### Model-Human Disagreement and Human Error Correction

Human errors in the annotation of message intents were inevitable given the dataset’s very large size. The data annotators were highly trained undergraduate students in public health fields who demonstrated high interrater reliability in training before annotating the study dataset. They met weekly with supervisors to resolve inconsistencies in real time. Despite this rigorous training and oversight, a small number of annotation errors were observed. Therefore, a domain expert thoroughly reviewed the model-human disagreements in the training, evaluation, and test sets to identify and correct human annotation errors.

Table 3 presents statistics on model-human disagreement on message intents from model mispredictions or human annotation errors or both. These statistics offer insights into the accuracy of human annotators and the potential of a large language model to sometimes outperform humans. Model-human disagreement was assessed in the downsampled datasets only, due to lack of resources to search for disagreement in the full dataset.

**Table 3.** Causes of model-human disagreement in the detection of intents in this study (United States).

Dataset (downsampled)	Model-human disagreement	Humans wrong, model correct	Model wrong, humans correct	Both humans and model wrong
Train	0.7%	59%	30%	11%
Evaluation	15%	42%	47%	11%
Test	15%	39%	55%	6%

In the downsampled training dataset (75% of the nontest data), the model and human annotators disagreed on the message intent 0.7% of the time. The human annotators were incorrect in 59% of these disagreed instances, with the model being accurate. The model mispredicted in 30% of the disagreed instances, with both human and model erroneous in the remaining 11% of the instances. Thus, in total, humans were wrong in 70% of the disagreed instances (59%+11%), amounting to 0.13% of the total data, 0.22% of the full training data, and 0.47% of the downsampled training data. So, human annotators were rarely wrong. While the downsized training dataset was not found to include many human annotation errors, it included some, indicating that retraining the model on a corrected dataset would lead to performance improvements.

In the downsampled evaluation dataset (25% of the nontest data, reduced further after downsampling the dominant off-topic intents), the model and human annotators disagreed on the message intent 15% of the time. Human annotators were wrong in 42% of these instances, with the model being accurate. The model mispredicted in 47% of these instances, and both were erroneous in the remaining 11% of the instances. Thus, in total, humans were wrong in 53% of the disagreed instances (42%+11%), amounting to 0.76% of the total data, 3.78% of the full evaluation data, and 8% of the downsampled evaluation data.

Finally, in the downsampled test dataset (holdout data from groups 30 to 36, reduced further after downsampling off-topic intents), model and human annotators disagreed on the message intent 15% of the time. Human annotators made errors in 39% of these cases, with the model being accurate. The model mispredicted in 55% of these instances, and both were erroneous in the remaining 6% of the instances. Thus, in total, humans were wrong in 45% of the disagreed instances (39%+6%), amounting to 0.50% of the total data, 2.5% of the full test dataset, and 7% of the downsampled test data.

Table 4 presents statistics on the corrections made to the downsampled data after manual review of the model-human disagreement in intent detection by category. In total, 1077 samples were corrected, accounting for 1.4% of the total dataset and 3% of the downsampled data, indicating human annotation errors and corrections were relatively rare. In the downsampled training dataset, in 102 cases, the support, smokefree, and greetings intents were corrected more frequently than other intents. In the downsampled evaluation dataset, in 588 cases, the support, smokefree, off-topic, cravings, and greetings intents were corrected more frequently than other intents. In the downsampled test dataset, in 387 cases, the smokefree, off-topic, quitdate, nrt_dontwork, nrt_itworks, and greetings intents were corrected more frequently than other intents.

**Table 4.** Data adjustments after reviewing model-human disagreement in smoking-cessation message intents in this study (United States).

Intent category	Adjustments to downsampled training dataset (n=102)	Adjustments to downsampled evaluation dataset (n=588)	Adjustments to downsampled test dataset (n=387)
cigsmell	0	3	5
cravings	9	43	14
ecigs	0	3	2
fail	1	23	7
greetings	10	53	43
health	1	26	18
nrt_dontwork	3	30	20
nrt_dreams	1	4	1
nrt_howtouse	0	21	10
nrt_itworks	3	17	15
nrt_mouthirritation	0	7	1
nrt_nauseous	0	9	5
nrt_od	0	5	3
nrt_skinirritation	0	3	3
nrt_stickissue	0	0	2
off-topic	10	71	33
quitdate	0	14	16
savingmoney	0	10	3
scared	3	6	9
smokefree	17	97	60
smokingless	0	10	1
stress	1	14	7
support	43	116	104
tiredness	0	2	2
weightgain	0	1	3

A domain expert handled the human annotation error correction and found that corrections were often needed when two intents were expressed in a single message. In such cases, the human annotators had been trained to prioritize certain intents over others to optimize the chatbot’s responses, but they apparently found this prioritization challenging. For instance, if a person said they quit but were craving, this should have been annotated as cravings, not smokefree, to prioritize a response to help with craving. If a person mentioned they were going to quit on day 1, this should have been annotated quitdate, not smokefree, to prioritize quitdate help. Messages about failing and setting a new quit date should have been annotated as fail, not quitdate, to provide guidance after a fail. If a message started with a greeting, for example, good morning, but then provided quitting support, it should have been annotated as support to prioritize a support-related response over a generic greeting. If a message discussed quitting but also reported an issue with NRT, it should have been annotated as NRT-related to assist with NRT use. If a person mentioned no longer needing NRT because they had successfully quit using NRT, it should have been annotated as nrt_itworks, not as a generic quit. All annotation corrections were conducted blind to the model predictions; that is, the domain expert reviewed and adjudicated the intent annotations independently of model outputs.

Overall, this analysis revealed the value of using large language models not only for classifying message intents but also for uncovering human errors in annotating these intents. The relatively high rate of model correctness in cases of human misannotations reinforces the idea that annotation corrections followed by model retraining can result in more accurate assessments of the model’s performance. However, in our work, the observed impact on model performance was minimized because we only had the resources to correct human annotation errors in the downsampled data, not the full data. Additionally, as human-model disagreement and human annotation correction often involved a message with two intents, different methods of addressing this situation should be considered, as will be discussed below.

## Discussion

### Principal Findings

Large language models have become the new state-of-the-art models in natural language processing due to their ability to recognize complex linguistic patterns and subtle semantic distinctions. Leveraging large language models for classification tasks like recognizing message intents can significantly enhance performance over earlier models. However, our analysis indicates that large language models may be insufficient for intent recognition in mobile health interventions when user messages are short and subtle wording differences can profoundly affect meanings. We studied messages in mobile health smoking-cessation support groups, where there was a profound difference between saying “quit” or “not yet quit,” “NRT works” or “NRT doesn’t work,” and “quit day 1” or “quit day 21,” and our off-the-shelf large language model was not always able to detect these nuances. The model’s performance was unacceptably poor until it was fine-tuned on a domain-specific dataset, which required laborious human annotation of message intents. The model before fine-tuning performed poorly because the domain-specific text often contained unique terminology, implicit meanings, and contextual subtleties that a general-purpose model was unable to fully grasp without exposure to relevant training data.

We found that fine-tuning the large language model on a domain-specific dataset led to substantial performance gains. The fine-tuning process allowed the model to familiarize itself with the characteristics of the dataset and better capture the intents behind the messages. Our fine-tuned large language model substantially outperformed the same model without fine-tuning. But a key challenge we identified during fine-tuning was data imbalance, particularly due to the dominance of off-topic message intents unrelated to the focal domain of smoking cessation. This imbalance caused the model to overpredict this majority (dominant) off-topic intent, degrading performance on all other intents, even though we assigned more importance to underrepresented categories when the model made mistakes. To address this issue, we implemented downsampling of the majority off-topic intent before fine-tuning, which increased unweighted *F*_1_-scores (overall performance) by 7% and 14% in the downsampled uncorrected dataset and downsampled corrected dataset, respectively, indicating a substantial improvement in balanced performance across all intents. Individual intent-level *F*_1_-scores also demonstrated consistent gains. When we evaluated our methods on the full uncorrected test dataset, the unweighted *F*_1_-score decreased when we added downsampling, primarily due to the large number of off-topic intents in that full dataset, which adversely affected precision. However, downsampling improved unweighted recall by approximately 10% in that full dataset, indicating more robust performance in correctly identifying in-domain, clinically relevant intents.

Another critical insight from our study was the identification of dataset inconsistencies and noise, largely arising from human annotation errors in intent labeling. To address this issue, we examined the model-human disagreement, identified human annotation errors, and corrected them. Even when the downsampled test dataset remained uncorrected, training on the corrected dataset slightly increased unweighted recall, suggesting improved generalization to noisy real-world data, and the *F*_1_-score was preserved. When the downsampled test dataset was itself corrected, the model trained with error correction achieved the same unweighted recall as no error correction, but the latter yielded approximately 3.5% higher precision, slightly improving the *F*_1_-score. This finding indicates that the no error correction method generalized marginally better to the corrected dataset than the error correction method.

This last outcome may indicate the error correction process, while beneficial for improving unweighted recall under noisy conditions, may have reduced useful diversity or led to minor overfitting. Another reason could be that most corrections involved the majority of intents such as support, greetings, and smokefree, and these samples were corrected into rarer intents. As a result, the error-correction method focused slightly more on the rare intent categories, achieving similar recall as the downsample-only method, but lower recall on the majority classes, reducing precision and the overall *F*_1_-score. On the full test dataset, the unweighted *F*_1_-scores of downsample-only and downsample with error corrections were comparable; however, error correction achieved a 2.5% higher unweighted recall and thus showed enhanced identification of in-domain intents under real-world conditions, where incoming data are noisy.

### Comparison With Previous Work

Previous work [20] studied intent classification with the same dataset, comparing random forest to BERT, which in 2018 was a revolutionary large language model introduced by Google. The study dataset, annotated by humans to indicate the different message intents, was identical to that used here, except the two smallest intent categories were combined and some duplicate messages were inadvertently retained. The findings showed that BERT, fine-tuned on the domain-specific annotated dataset, outperformed random forest. BERT yielded unweighted and weighted *F*_1_-scores of 0.67 and 0.84, respectively, which were reasonable scores but inadequate for implementation of the large language model in our mobile health intervention. In this study, we used a more recent public-domain large language model, Llama-3 8B (Meta), to perform intent classification across 25 message intent categories. When fine-tuned on the same domain-specific annotated dataset, Llama-3 8B achieved unweighted and weighted *F*_1_-scores of 0.72 and 0.86, respectively, on the full test dataset, outperforming the previously reported BERT model by 7% in unweighted and 2% in weighted *F*_1_-scores, respectively. However, these improvements should be interpreted as indicative rather than strictly comparable, due to differences in dataset structure and inadvertent duplication in the prior study.

We then evaluated our last two methods: (1) fine-tuning with downsampling and (2) fine-tuning with downsampling and error correction. When assessed on the downsampled corrected test dataset, these approaches achieved unweighted *F*_1_-scores of 0.88 and 0.86 and weighted *F*_1_-scores of 0.91 and 0.90, respectively. Relative to the BERT baseline, these are improvements of 31% and 28% in unweighted *F*_1_-scores and 8% and 7% in weighted *F*_1_-scores. We note that these improvements are only indicative, since differences in dataset prevent a strict empirical comparison. However, when evaluated on the full test dataset, our final two methods achieved unweighted *F*_1_-scores of 0.58 and 0.57, and weighted *F*_1_-scores of 0.66 and 0.65, lower than the previously reported BERT performances. This reduction is primarily attributable to the dominance of the off-topic intent category in the full test dataset. Misclassification of off-topic intents as in-domain intents reduced overall precision and the resulting *F*_1_-scores. Despite reduced precision, our final two methods achieved substantially higher unweighted recall scores of 0.80 and 0.82, compared with 0.71 for the prior work, representing a 13%‐16% recall improvement. This finding indicates that our approach is more effective at identifying in-domain, clinically meaningful intents, which is our central objective. However, our model’s weighted *F*_1_-score of 0.65 is substantially lower than the previously reported 0.84 for the BERT baseline. This indicates a clear reduction in overall precision and *F*_1_-score performance in a real-world setting, highlighting the trade-off between improved recall for in-domain intents but decreased overall classification accuracy. In addition, a strict comparison with the previous work is not feasible because it used 24 intents after merging two small ones (instead of our 25) and inadvertently contained duplicated data. These differences alter the ground truth distributions and, therefore, the performance differences should be interpreted as observed rather than strictly comparable.

### Implementation Feasibility, Generalizability, and Potential Risks

Fine-tuning the large language model based on our final method took approximately 2.5 hours, and each input was processed in approximately 40 milliseconds, with a peak GPU memory usage of 8.2 GB. The model was able to handle approximately 493 tokens per second, demonstrating fast and efficient performance suitable for real-time intent detection. Human oversight was incorporated for human annotation error correction, to improve model accuracy and reliability. This error correction approach was straightforward. Model-human inconsistencies in intent classifications were identified and then systematically reviewed to identify potential human annotation errors, with error corrections performed by a domain expert who was blind to the model predictions. If a domain expert with knowledge of the annotation process or domain context is involved, this error-correction approach should be reproducible.

Regarding the generalizability of our final model, it should perform well on new data that mainly includes the same 25 intent categories. Large language models handle shifts in language use through embedding-based representations, which capture semantic meaning rather than relying on exact word sequences. Within the same domain, this approach mitigates the impact of data drift, ensuring that the model’s predictions remain relevant and accurate even if specific word usage has evolved over time. However, if the new data contain different intent categories, it would require reannotation or model adaptation.

The largest failure in our intent-detection model would be if an intent category is detected as a different and unsuitable intent category. For instance, the message “Started my 5th day the struggle is real today” refers to cravings, but the model might mispredict it as smokefree. While it is true that on the 5th day the person is smokefree, the main concern is cravings as there is struggle, so missing the craving intent may prevent the chatbot from responding with the needed support for cravings. Another example is that the message “I’m restarting Friday” refers to fail, but the model might mispredict it as quitdate. Although the message mentions Friday as the quit date, it mainly indicates the person failed to quit and needs help with avoiding a future failure, not with setting a quit date. These examples highlight limitations in intent detection and suggest that our future work should aim to provide appropriate responses even if the predicted intent is incomplete or erroneous or multiple intents are conveyed in a single message.

In our analysis, we downsampled the dominant off-topic intent category to help the model better learn the remaining 24 in-domain intent categories. As a result, the model’s performance on off-topic is lower, and these messages are more likely to be misclassified as one of the in-domain categories. In practice, this means that some off-topic messages may receive an in-domain, prevetted smoking-cessation response. Although off-topic messages typically fall outside the scope of smoking cessation and may include general social conversation, misclassifying them as cessation-related is unlikely to cause harm as people signed up for a smoking-cessation support group. Moreover, cessation responses are empathetic, supportive, and encouraging, and therefore still appropriate in tone. However, this high rate of false positives should be considered a potential barrier to user adoption and trust, rather than inherently unproblematic, as we do not have supporting user data to assess its real-world impact.

We acknowledge that repeated false-positive delivery of cessation-oriented content to users engaging in social small talk may risk user disengagement. From a deployment perspective, we consider two complementary definitions of signal-to-noise. When true in-domain detections are considered signal and off-topic detections are considered noise, based on our full test dataset, our downsampling methods would result in nearly 4700 off-topic messages being misclassified as in-domain, compared to 5500 that are truly in-domains. This corresponds to nearly one unnecessary cessation intervention for every appropriate one, resulting in roughly a 1:1 signal-to-noise. Where both true in-domain detections and off-topic detections are considered signals because off-topic messages appropriately redirect people to focus on the smoking-cessation domain, our methods would result in nearly a 2:1 signal-to-noise. In sum, in the real deployment, the model could send from 1 to 2 useful cessation messages for every unnecessary one. This means users engaging in casual or social conversation could frequently receive irrelevant intervention messages. This potential risk should be continuously monitored during real-world deployment to mitigate unintended user disengagement.

It is true that recognizing off-topic intent messages is an important goal for this deployment, as detecting such messages can possibly help redirect focus in the smoking-cessation domain. However, detecting in-domain intent categories is a higher-priority objective. The original dataset contained a large proportion of off-topic messages, which biased the model toward off-topic detection and, in turn, reduced performance on in-domain messages. To address this tradeoff, we prioritized in-domain detection by weighting it more heavily, while still considering off-topic detection as a secondary goal. Consequently, in our downsampling approach, we reduced the number of off-topic messages to improve the model’s performance on in-domain intent categories.

### Limitations

Our final method of large language model training that included fine-tuning, downsampling, and error correction achieved impressive performance in our dataset, but it had several limitations. First, a continued challenge is complex messages involving two or more intent categories. For instance, a user stated, “Tomorrow is my day 1 and I haven’t smoked for over 5 hours today,” which reflects two intents: quitdate (planning to quit tomorrow, ie, day 1) and smokingless (starting to quit but still smoking some). While the main message intent was likely to alert others to tomorrow being the quitdate, the model predicted smokingless. Similarly, in “I’m not going to quit smoking on my busiest day,” the message expressed intent to quit on a specific day (quitdate) but also implied being smokefree on that day; however, the correct intent category was quitdate and the model mispredicted it as smokefree. These examples highlight the inherent ambiguity in single-intent annotations when messages express multiple intents. Because the dataset does not account for multi-intent expressions, the model’s predictions occasionally diverged from the human annotations despite both being semantically reasonable.

Another study limitation was that 2 of our 4 methods involved downsampling the off-topic intent category in the training, evaluation, and test datasets, based on the average number of samples of all other intents. Downsampling the off-topic intent category consistently across datasets ensured the model learned from a representative distribution of focal intents. However, we recognize that altering the test dataset may be problematic. Therefore, we also evaluated performance on the complete, uncorrected test dataset which likely offers a more accurate reflection of how the model would perform in real-world scenarios.

A third potential limitation of our study is that, in our final method, we corrected human annotation errors. However, we only had the resources to assess the much smaller downsampled dataset for annotation errors, and we only examined instances where the model and the human annotator disagreed. This approach introduces the risk of circular bias, where the ground truth is shifted toward the model’s predictions. This may inadvertently penalize natural human variation and inflate performance metrics relative to what the model would achieve on truly independent unmodified data. Moreover, since our error correction method focused only on messages where the model disagreed with human annotations, instances where both the model and human annotators were incorrect (shared errors) were not reviewed, which may leave residual annotation errors in the dataset. As a result, even the corrected dataset may be partially aligned with the model’s predictions rather than fully independently verified, which could influence performance estimates. Once again, performance on the full uncorrected test dataset likely provides a more realistic estimate of generalization to real-world conditions.

Fourth, because the training and evaluation data splits were stratified based on the distribution of intent categories rather than at the group or user level, messages from the same user could appear in both datasets, which may introduce data leakage and allow the model to partially learn user-specific writing styles, potentially overestimating performance.

Fifth, while we downsampled the dominant off-topic intent, the dataset remained imbalanced. Rare intents still had very few examples, whereas intents such as greetings and support had many examples, which reduced model performance for underrepresented intents. Finally, our off-the-shelf model with no fine-tuning produced 991 intent predictions that did not map onto any of our existing intent categories. To handle this, we included these samples as misclassifications in the final scores.

### Future Work

In future work, we plan to explore how to address multiple intents in messages, for instance, by allowing the model to detect the most probable 2‐3 intents. We will also explore ways to incorporate general domain knowledge into the model training process, for example, patterns or semantic rules that are not easily learnable from data alone, but can be integrated into the model’s decision-making. We may also seek to expand our dataset by collecting additional real-world examples of underrepresented intent categories and/or creating synthetic examples using generative artificial intelligence (AI). However, it will be crucial to ensure that any synthetic data mimics real-world patterns and does not introduce bias. In addition, we may explore temporal analyses using classifiers to address possible temporal variability in the data.

In additional future work, we will explore threshold tuning. For this study, we did not perform threshold tuning or probability calibration because the Llama-3 8B model inherently selects the most probable category based on its prediction probabilities. We were concerned that adjusting thresholds could suppress predictions for certain intent categories when confidence scores were low. In our domain, where many intents are semantically related, the model must still assign a category even with lower confidence to remain contextually helpful. Setting a high threshold might cause the model to abstain from predicting any intent, which would be less useful to us. Although a low-confidence prediction may occasionally be incorrect, it should still tend to remain on-domain and informative. Notwithstanding, threshold tuning should be considered. Specifically, the off-topic intent category can be almost 50% misclassified as on-topic or domain-related, leading to on-topic chatbot responses, and this could cause user disengagement. So, future work should explore confidence-based thresholding or probability calibration to reduce false-positive domain-related chatbot responses. By implementing a mechanism to silence the chatbot when the predicted intent probability is low (eg, not likely to be on-topic), the signal-to-noise ratio could be improved in live deployment. This approach may help minimize unnecessary domain-specific cessation messages, reduce user disengagement, and enhance overall safety and usability in real-world clinical settings.

We chose the arithmetic mean as a simple and transparent method to reduce the dominance of the off-topic intent category while retaining sufficient examples of it for model learning. Using the mean ensured that the downsampled off-topic intent remained large enough to capture diversity within its messages. But we acknowledge that downsampling to the mean likely resulted in a reduced off-topic intent category that was still significantly larger than many minority intent categories. Thus, alternative strategies such as downsampling to the median could be explored in future work.

Our ultimate research aim is to create an intent detection model that will also respond to messages based on the intents conveyed. Therefore, we still need to create the model’s response capabilities, yielding a complete interactive chatbot. We hope this chatbot will improve engagement in our smoking-cessation support groups by ensuring at least the chatbot is available to respond 24/7 if no human is online. However, we acknowledge that our mobile health support group intervention is not a one-size-fits-all approach; for instance, it is not suitable for people who are not interested in mobile health and seek in-person support exclusively.

To translate technical performance into operational impact, we hope to test our chatbot in a randomized controlled trial to see if it significantly improves the outcomes of our mobile health intervention for smoking cessation. We plan to assess the potential lift in engagement due to the inclusion of a chatbot that accurately detects and responds to support group members’ messages. We also plan to assess other intervention outcomes, including cessation success.

### Conclusions

Mobile health smoking-cessation interventions that provide real-time supportive interactions have the potential to deliver timely advice and improve health outcomes. Detecting user intents from their utterances can enhance personalization and engagement by identifying individual needs and enabling intelligent responses through chatbots, that is, AI agents. However, existing chatbots for smoking-cessation interventions are limited in their intent detection and response capabilities. In this study, we developed a large language model for intent detection and evaluated its performance under multiple conditions: using an off-the-shelf model without fine-tuning, fine-tuning on domain-specific data, fine-tuning with downsampling of the dominant off-topic intent category, and fine-tuning with downsampling combined with correction of human annotation errors identified from model-human disagreement. The off-the-shelf and fine-tuning–only methods demonstrated poor performance in detecting in-domain intents. Fine-tuning with downsampling achieved unweighted and weighted *F*_1_-scores of 0.88 and 0.91, respectively, on the downsampled corrected dataset, and unweighted and weighted *F*_1_-scores of 0.58 and 0.66 with an unweighted recall of 0.80 on the full test dataset. Downsampling with error correction achieved 0.86 unweighted and 0.90 weighted *F*_1_-scores on the downsampled corrected test data, and 0.57 unweighted and 0.65 weighted *F*_1_-scores on the full test dataset. Downsampling the training dataset lowered precision and therefore the *F*_1_-scores on the full dataset due to the high prevalence of the off-topic intent category in the latter. However, downsampling with error correction achieved slightly higher unweighted recall (0.82) on the full test dataset compared with downsampling alone (0.80), indicating only modest improvement in detecting in-domain message intents. This finding suggests the error correction could conceivably enhance performance; though the difference is small, it is statistically significant. Thus, automated downsampling alone may provide sufficient benefit, though the resource-intensive manual error correction process could offer slightly better returns in practice.

## Supplementary material

10.2196/83437 Multimedia Appendix 1 Supplementary tables and figures illustrating the study’s evaluation process.

## Abbreviations

8B: 8 billion parameters
AI: artificial intelligence
BERT: Bidirectional Encoder Representations From Transformers
Bi-LSTM: bidirectional long short-term memory
BLEU: Bilingual Evaluation Understudy
CPU: central processing unit
CUDA: Compute Unified Device Architecture
EDA: easy data augmentation
EOS: end-of-sequence
FP32: 32-bit floating point
GPU: graphics processing unit
HS: human subjects
IRB: institutional review board
LoRA: low-rank adaptation
LSTM: long short-term memory
MIMIC-III: Medical Information Mart for Intensive Care-III
NRT: nicotine replacement therapy
PEFT: parameter-efficient fine-tuning
QLoRA: quantized low-rank adaptation
SVM: support vector machine
UC: University of California
UCI: University of California, Irvine
