# Supplementary material for: Enhancing Detection of Message Intents in a Mobile Health Smoking-Cessation Intervention Using Large Language Model Fine-Tuning, Data Downsampling, and Error Correction: Algorithm Development and Validation
Source: J Med Internet Res. 2026 Mar 9;28:e83437. doi: 10.2196/83437 (PMC12978910; doi:10.2196/83437)
Supplement: Multimedia Appendix 1 [file jmir-v28-e83437-s001.pdf]

This is a Multimedia Appendix to a full manuscript published in the J Med Internet Res. For full copyright and citation information see http://dx.doi.org/10.2196/jmir.83437

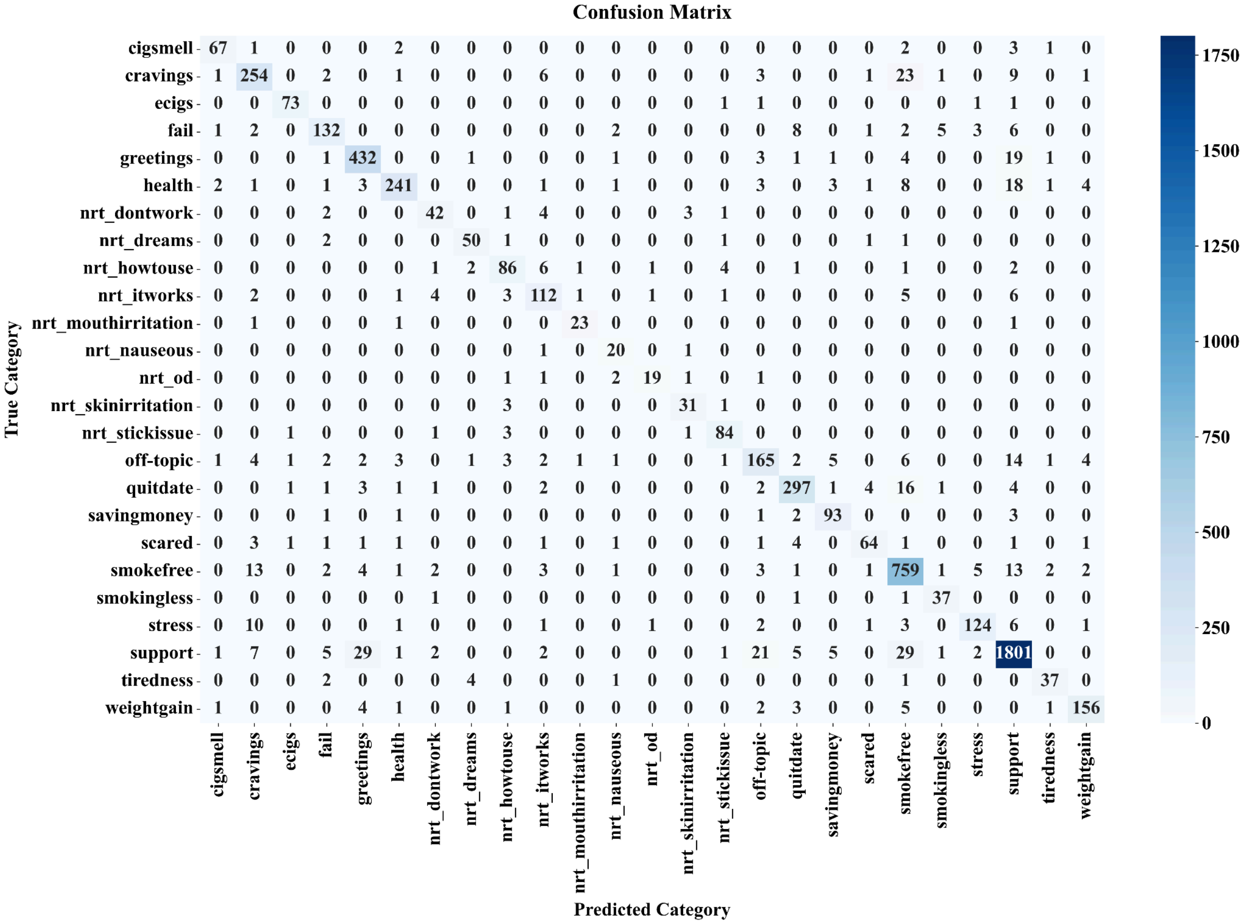

Figure S1. Confusion Matrix of all Intent Categories for Large Language Model with Fine-tuning, Downsampling, and Error Correction on Downsampled and Error Corrected Test Data.

In Figure S1, diagonal entries in the matrix indicate the number of correctly predicted instances for each category, while the off-diagonal entries show the misclassifications between categories. From the confusion matrix, we can observe specific patterns of confusion across intent categories. For instance, the "cravings" category was misclassified as "smokefree" 23 times, and "fail" was confused with "quitdate" 6 times. Additionally, intents such as "greeting," "stress," "health," "off-topic", and "smokefree" were frequently misclassified as "support." These patterns align with the trends observed in Figure 8 of the main manuscript.

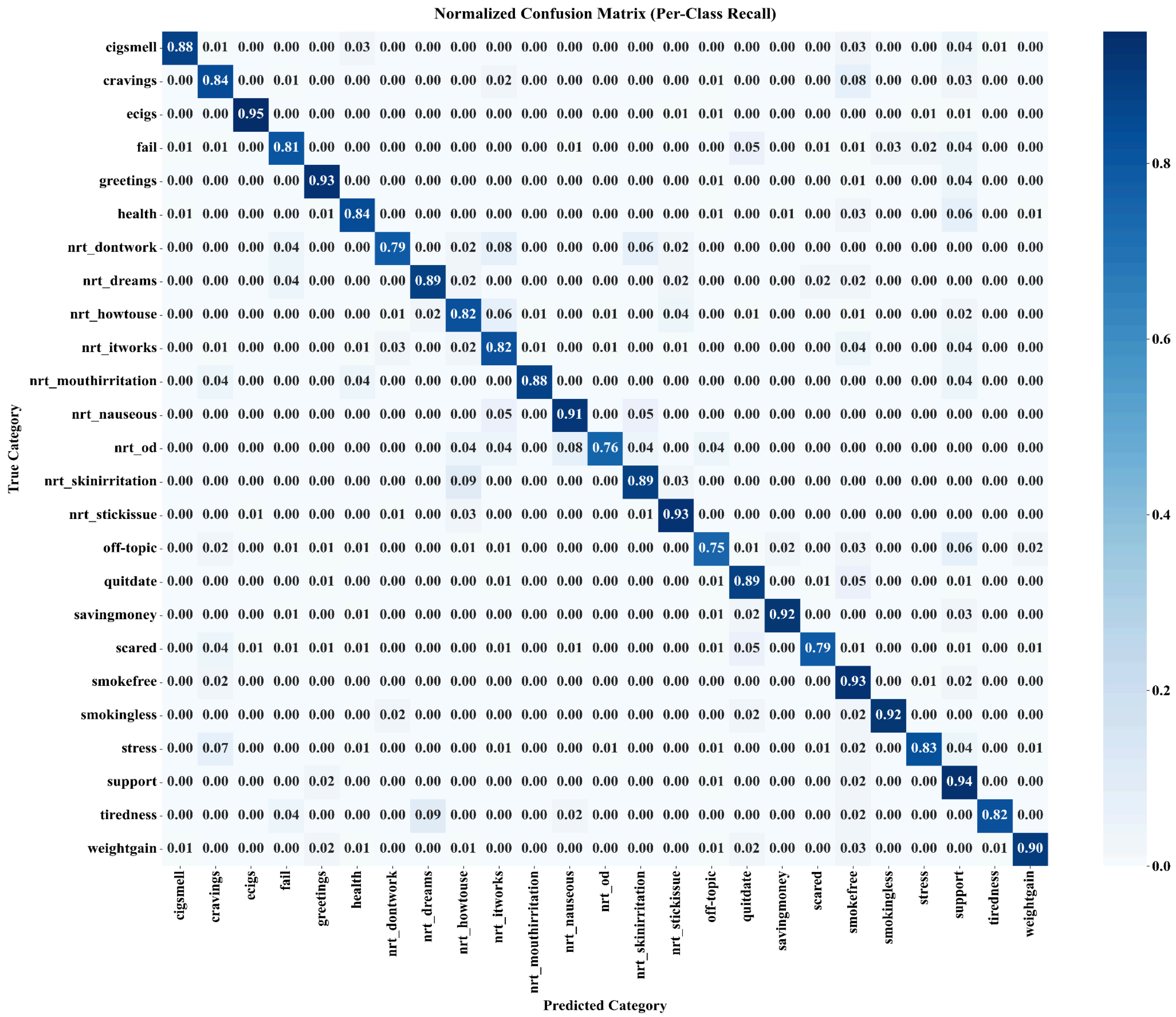

Figure S2. Normalized Confusion Matrix (Per Class Recall) of all Intent Categories for Large Language Model with Fine-tuning, Downsampling, and Error Correction on Downsampled and Error Corrected Test Data.

We present Figure S2 because, while Figure S1 displays the raw counts of correctly and incorrectly classified samples, it does not accurately reflect performance trends due to the category imbalance in the dataset. Normalizing the confusion matrix by the total number of samples per category provides a clearer visualization of each class's relative performance. In Figure S2, the deep blue diagonal cells represent high per-class performance, illustrating how well each intent category was correctly identified. The few off-diagonal lighter cells indicate instances of misclassification, consistent with earlier observations. For example, the "cravings" category was occasionally misclassified as "smokefree", and "fail" was confused with "quitdate." Similarly, intents such as "greeting," "stress," "health," "off-topic," and "smokefree" were frequently misclassified as "support."

Table S1. Precision, Recall, and F1-scores of Different Downsampling Setups of Dominant Off-Topic Category for Large Language Model with Fine-tuning and Downsampling on the Evaluation dataset.

**"Off-topic" Category Downsampling Type**	**Unweighted Precision**	**Unweighted Recall**	**Unweighted F1-score**	**Weighted Precision**	**Weighted Recall**	**Weighted F1-score**
Average of top 3 most frequent categories excluding "off-topic"	0.79	0.78	0.78	0.81	0.81	0.81
Average of top 12 most frequent categories excluding "off-topic"	0.79	0.79	0.77	0.83	0.83	0.83
Average of top 13 most frequent categories excluding "off-topic"	0.80	0.79	0.79	0.84	0.84	0.84
**Average of all other categories excluding "off-topic"**	**0.80**	**0.79**	**0.80**	**0.85**	**0.85**	**0.84**

Table S1 presents the precision, recall, and F1-scores for different downsampling setups of the "off-topic" intent category for the large language model with fine-tuning and downsampling on the evaluation dataset. We experimented with four downsampling strategies which are downsampling it to mirror the average sample size of the: (1) top 3 most frequent categories except "off-topic", (2) top 12 most frequent categories except "off-topic", (3) top 13 most frequent categories except "off-topic", and (4) all categories except "off-topic".

Among the evaluated configurations, the setup that used the average of all categories except "off-topic" achieved the best overall performance, with an unweighted precision of 0.80, recall of 0.79, and F1-score of 0.80 and weighted precision and recall reaching 0.85 and weighted F1-score reaching 0.84. Additionally, maintaining a smaller proportion of "off-topic" messages ensures the number of instances requiring expert review after human-model disagreement remains within a feasible range. Therefore, we selected this configuration as the final downsampling version for subsequent analyses.

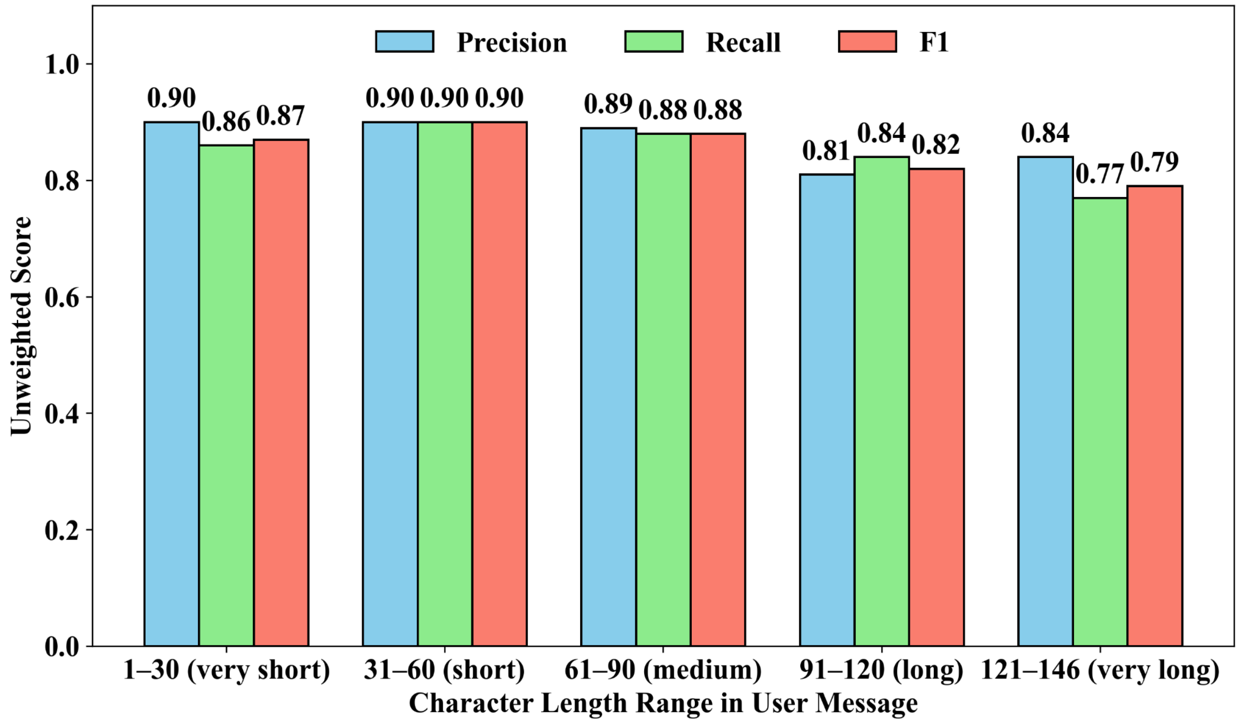

Figure S3. Comparison of Precision, Recall, and F1-score based on Message Character Lengths for Large Language Model with Fine-tuning, Downsampling, and Error Correction.

In Figure S3, the downsampled test data were divided into five message length categories: very short (1–30 characters; 747 samples), short (31–60 characters; 1,369 samples), medium (61–90 characters; 1,170 samples), long (91–120 characters; 2,177 samples), and very long (121–146 characters; 323 samples). As shown in the figure, performance remains strong for very short to medium-length messages, with precision, recall, and F1-scores around 0.88. However, these scores decline to approximately 0.80 for longer messages, suggesting that model performance decreases as message length increases, likely because longer texts introduce more complexity or multiple intents, making them harder for the model to classify accurately.
